# Key Design Features of Lipid Nanoparticles and Electrostatic Charge-Based Lipid Nanoparticle Targeting

**DOI:** 10.3390/pharmaceutics15041184

**Published:** 2023-04-07

**Authors:** Vijay Gyanani, Roshan Goswami

**Affiliations:** 1 T.J.L. School of Pharmacy, University of the Pacific, Stockton, CA 95211, USA; 2 Intas BioPharmaceuticals, Ahmedabad 382213, India

**Keywords:** lipid nanoparticles, LNP design, LNP potency, LNP immunogenicity, LNP biodegradability, pKa tunability, lipid tail design, lipid headgroup design, pH-sensitive liposomes, convertible liposomes

## Abstract

Lipid nanoparticles (LNP) have gained much attention after the approval of mRNA COVID-19 vaccines. The considerable number of currently ongoing clinical studies are testament to this fact. These efforts towards the development of LNPs warrant an insight into the fundamental developmental aspects of such systems. In this review, we discuss the key design aspects that confer efficacy to a LNP delivery system, i.e., potency, biodegradability, and immunogenicity. We also cover the underlying considerations regarding the route of administration and targeting of LNPs to hepatic and non-hepatic targets. Furthermore, since LNP efficacy is also a function of drug/nucleic acid release within endosomes, we take a holistic view of charged-based targeting approaches of LNPs not only in the context of endosomal escape but also in relation to other comparable target cell internalization strategies. Electrostatic charge-based interactions have been used in the past as a potential strategy to enhance the drug release from pH-sensitive liposomes. In this review, we cover such strategies around endosomal escape and cell internalization in low pH tumor micro-environments.

## 1. Introduction

Lipid nanoparticles (LNPs) have emerged as a critical tool in the treatment of genetic/hereditary disorders, infectious diseases, and cancers, among other illnesses. LNP systems have been established to yield a safe and efficacious delivery of nucleic acid and small molecule drugs to target cells in the body. The worldwide use of mRNA COVID-19 vaccines has ensured wide acceptability within scientific and regulatory bodies. Such systems have therefore drawn immense attention from academia and industry alike. For example, a search on clinicaltrials.gov reveals a sizeable number of ongoing clinical trials on LNP products. A list of these trials is presented in Table 1. 

As seen from the sheer number of currently ongoing clinical studies (Table 1), LNPs are a field of “hot pursuit” for the delivery of nucleic acids, small molecules, and other drug cargos. This highlights the importance of gaining insights into LNP drug delivery systems. 

Lipid-based delivery systems have come a long way over several decades since their first discovery and use as liposomes, which are lipidic bilayer vesicles broadly encapsulating hydrophobic drugs in the bilayer and hydrophilic drugs in the vesicular core. Liposomes are formed by liquid–crystalline lipid bilayers and have been prepared by complex production methods. To simply the manufacturing process and add rigidity to lipid vesicles, solid lipids were incorporated into the system to form solid lipid nanoparticles. Mixtures of solid and liquid crystalline lipids were used to form nanostructured lipid carriers. Then, with the requirement of incorporating a charged drug cargo, counter-charged lipid exploration was initiated in lipid nanostructured delivery systems and the need for less toxic nanocarriers brought about the use of ionizable lipids. The quest for an enhancement in endosomal escape brought about the use of lipids with inverted cubic and hexagonal liquid crystalline phases. The progress of lipidic nanocarriers to the current form of LNPs, which are the most sought after lipidic nanocarriers for clinical use nowadays, has been recently thoroughly reviewed by Tenchov et al. [1]. Overall, lipidic nanocarriers have been extensively explored, from their conventional uncharged vesicular form to the present day ionizable lipid core LNPs. In the present review, for simplicity of understanding, the general inherent principle of a bilayer surface is used in the figures to explain nanoparticle design elements and the way they work.

Herein, we discuss some key design principles of LNPs. Specifically, we discuss the potency, biodegradability, and immunogenicity of LNP systems, which are important considerations for developing such systems. The basic design aspects of LNPs presented in this review hold true regardless of the type of drug cargo, i.e., macromolecules or small molecules. 

Moreover, the route of administration and targeting of LNPs to hepatic and non-hepatic targets depend on some key underlying considerations. We cover the rationale regarding the selection of the route of administration. We also briefly discuss targeting approaches to non-hepatic targets. 

Besides the above-mentioned product characteristics, one of the critical hurdles, however, with intracellular delivery of LNPs is the release of the drug cargo from the endosome [2]. It has been estimated that only <2% of API escapes the endosome intracellularly [3]. One of the core focus areas of LNP development, therefore, is the better design of these systems to enhance endosomal drug escape. Such LNP design strategies revolve around enhanced electrostatic charge interactions with the endosome and subsequent LNP destabilization to release the drug cargo. 

The electrostatic-charge-based drug delivery mechanism has been an integral part of pH-sensitive liposomal drug delivery systems as well. pH-sensitive liposomes are strikingly similar to LNPs in that they contain pH-sensitive lipids akin to the ionizable pH-sensitive lipids in LNPs. While ionizable lipids protonate in a low pH environment, pH-sensitive lipids can have different mechanisms to trigger drug release. They can either (i) protonate in mildly acidic environments and acquire positive charges akin to ionizable lipids or (ii) have pH-labile groups that cleave or change conformation under such low pH environments that destabilize the LNPs. Such pH-sensitive lipids therefore help enhance the charge-based uptake of liposomes in target cells or trigger cargo release by destabilizing liposome membranes in a manner similar to ionizable lipid-induced destabilization in LNPs. Herein, we discuss such electrostatically triggered drug targeting strategies of LNPs and similar attempts made to develop such strategies with pH-sensitive liposomes for endosomal escape and tumor targeting. We first focus on the key aspects of the design principles of LNPs and subsequently cover different charged-based targeting approaches. 

As part of the literature search methodology for this article, we reviewed recent approaches to modulating the ionizable lipid pH tunability, to lipid tail designs of constituent lipids, and to the biodegradability and immunogenicity of LNP systems. We also reviewed relevant electrostatic charge-based targeting strategies of LNPs. 

## 2. LNP Design Principles

The therapeutic potential of a nucleic-acid-loaded LNP is dictated by its potency, biodegradability, in vivo distribution in the target tissue, safety, and immunogenicity. All these criteria are fulfilled by the efficient design of LNPs, i.e., careful choice of ionizable and other lipid constituents. 

The following are key design parameters for LNPs discussed in this review:

- Potency;
- Biodegradability/Tolerability;
- Immunogenicity.

### 2.1. Potency

The potency of an LNP is decided by its effectiveness in extravasation in the target organ and in internalization and subsequent discharge of cargo in the target cells. An LNP system essentially comprises an ionizable lipid, a helper lipid, cholesterol, and a polyethylene glycol (PEG) lipid. The surface charge and shape of the lipid components of a nanoparticle are indispensable features that enable the potency of an LNP system. The surface charge is influenced by the charged lipids on the LNP surface. 

It is well documented that the net surface charge on the LNP surface is known to trigger the reticuloendothelial system (RES) and cause rapid blood clearance [4]. Ionizable cationic lipids are designed such that they remain neutral at physiological pH and acquire a positive charge by protonating in a low pH endosomal environment to assist in endosomal escape of the drug load.

#### 2.1.1. Ionizable Lipid Design

The design of ionizable lipids has two hallmark features: (i) pKa of the headgroup that renders it largely neutral charge at physiological pH and allows for protonation in low pH environments in endosomes and (ii) design of the lipid tail that renders it more fusogenic and allows for escape of the cargo from the endosome. 

Both Pfizer and Moderna COVID-19 vaccines have amino alcohol lipid head groups and have demonstrated their efficacy and safety clinically. The pKa and other information on FDA approved RNA LNP products are provided in Table 2. 

##### Lipid Headgroup Design—pKa Tunability

The pKa of the ionizable lipids depends on the head group chemical composition, i.e., imidazole, piperazine, tertiary amine (Patisiran), and amino alcohol (Pfizer and Moderna), the presence of esters, the presence of linkers between the head group and the lipid tail, branching in the lipid tails, etc. A snapshot of such pKa-tunable features imparted by the head group chemistry are discussed below.

One functional group that alters the pKa of the ionizable lipid is an ester group. Sabnis et al. [7] synthesized a series of amino alcohol ionizable lipids. They concluded that the removal of one ester group from lipids with two ester groups in the lipid tail resulted in a reduction in the pKa. They also showed that adding carbon chain substitutes around the ester group in the lipid tails further reduced the pKa. In a similar study, Maier et al. [8] showed that as the ester group in the lipid chains moved away from the tertiary amine head group, the basicity decreased. Overall, it was demonstrated that the ester group affected the pKa and that lipid tunability was achieved by varying the distance of this functional group from the lipid head group. 

Additionally, no change in pKa was observed when alcohol functionality in the head-group was replaced with dimethyl amine [7].

Moreover, Huang et al. [9] prepared imidazole-based ionizable lipids and showed the dependence of the pKa on the methyl group substitutions on the imidazole moiety. As the number of methyl group substitutions increased from 0 to 2, the pKa increased from 5.53 to 6.75. The relative increase in pKa by addition of methyl groups can be attributed to the inductive effect of the methyl group on the imidazole nitrogen, which pushes its pKa to the basic side. 

In a different approach, linker groups were placed between the head group (either a tertiary amine or a piperazine) and the lipid carbon chains, and their effect on pKa and potency was studied [10]. It was concluded that hydrazine linkers imparted a higher pKa to ionizable lipids compared to either hydroxyl amine or ethanolamine linkers. They also showed that internalization in the multiple myeloma cell line was more for LNPs with ethanolamine linkers as opposed to hydroxyl amine linkers.

Besides the lipid pKa, the head group type also seems to have an impact on LNP internalization. Ni et al. [11] also prepared a series of piperazine-based LNPs where an ester group was eliminated and an amide group was introduced into the ionizable lipid design. A high mRNA transfection in non-hepatocytes immune cells, i.e., Kupffer cells, spleen macrophages, etc., were observed. Interestingly, Ramishetti et al. [10] concluded that LNPs with a piperazine headgroup accumulated more in the spleen and liver as opposed to lipids with a tertiary amine headgroup, which accumulated more in the liver when LNPs were injected intravenously in mice. Additionally, hydroxylamine and ethanolamine linker lipids provided better gene silencing in lymphocytes than hydrazine linkers [10].

##### Lipid Tail Design

The lipid headgroup design detailed hitherto provides critical tunability to the lipid and allows for the generation of positive charges in the endosomal microenvironment. However, this only serves as a precursor to the subsequent interaction of LNPs with the endosomal membrane. An efficient lipid tail design facilitates further engagement with the endosomal membrane and the release of drug cargo. Lipid tails can be designed to serve two functions: (i) make lipids fusogenic to allow for efficient endosomal escape or (ii) better pack lipid constituents within the LNP membranes to allow for effective drug retention while the LNP is in systemic circulation. 

**Figure 1:**
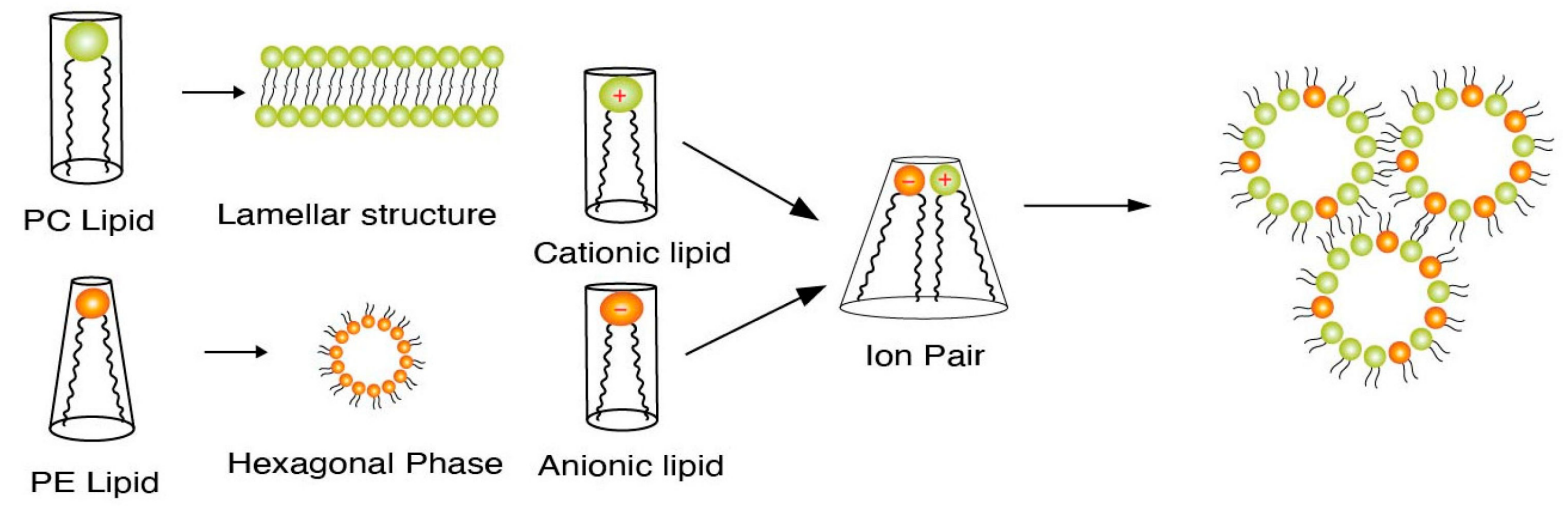
Figure depicting ion pair formation and subsequent formation of a hexagonal phase as a precursor to the assimilation/disruption of the endosomal membrane [15].

**Figure 2:**
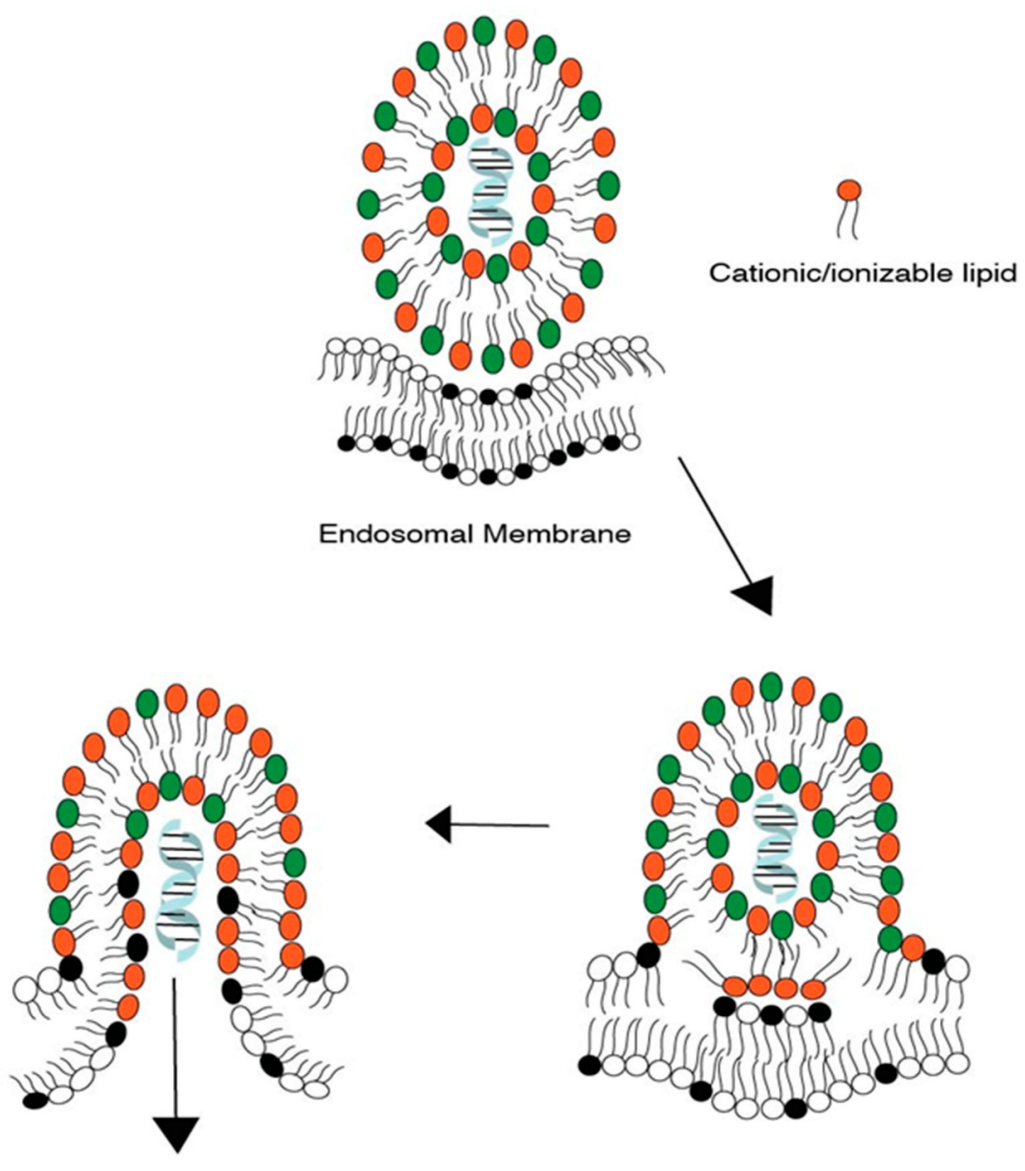
Release of cargo from an LNP after hexagonal phase formation and the subsequent assimilation/disruption of the endosomal membrane. Lipids with red head-groups represents ionizable lipids; lipids with green head-groups represent helper lipids and lipids with black head-groups represent negatively charged lipids on endosomal membrane. Drug cargo is represented by helical structure.

Endosomal escape is facilitated by the lipid tail as well as the presence of helper lipids. We first discuss the process of endosomal escape. Specifically, ionizable lipids acquire a positive charge in a low pH endosomal environment and interact electrostatically with the anionic lipids present in the endosomal membrane. Such interactions generate cationic–anionic ion pairs, which in turn allow for the transition from the lamellar phase to the inverted hexagonal phase, triggering delivery of cargo into the cytoplasm [12,13,14]. Figure 1 demonstrates such transitions from the lamellar to the hexagonal phase upon formation of ion pairs [15]. This phenomenon of a geometric transition from the lamellar to the hexagonal phase is controlled by the intrinsic curvature of the lipid components of LNPs. In a controlled experiment by Hamai et al. [16], it was determined that lipids with a low intrinsic curvature tended to form hexagonal structures as opposed to lipids with higher intrinsic curvatures. The intrinsic curvature is explained by the shape of lipids in that the more cylindrical-shaped lipids, e.g., phosphatidylcholine (PC) lipids, tend to form lamellar structures, while conical helper lipids, such as phosphatidylamine (PE), form hexagonal structures. This concept of intrinsic lipid curvature can be easily extended to intrinsic curvature imparted by lipid tail design of the ionizable lipids and the presence of helper lipids. Lipid tails designed with an increased cross-section have an increased intrinsic curvature of the lipid molecule, e.g., cone-shaped lipids. This increase in intrinsic curvature leads to better fusogenicity with the endosomal membrane. In an LNP system, once the ion pairs are formed upon protonation of the ionizable lipid, the geometric transition to a non-bilayer structure is aided by the presence of “high intrinsic curvature” helper “PE” lipids and cholesterol. Helper lipids also provide increased fluidity to the nanoparticle structure [17,18]. The formation of a hexagonal structure is a precursor to the disruption/assimilation of the endosomal membrane and the release of cargo from LNPs, see Figure 2. 

Lipid tails, therefore, can be designed to trigger the formation of hexagonal phases and the disruption of the LNP membrane. 

##### LNP Fusogenicity

Lipid designs that impart more fusogenic character to the LNP are (i) unsaturation in the lipid tails, e.g., patisiran ionizable lipid [5,19], (ii) branching in the lipid carbon chains as shown in the Pfizer and Moderna COVID-19 LNP ionizable lipid, (iii) multi-tail lipids, e.g., A9 by Acuitas and TT3 lipids [20], and (iv) polymeric lipids, e.g., 7C1 and G0-C14 lipids [21]. 

Unsaturation in the lipid tail introduced by incorporation of double bonds in the lipid tail provides fluidic character to the lipid and facilitates fusion with the endosomal membrane. Heyes et al. [22] established that by increasing the number of double bonds in the lipid tails from 0 to 2, the fusogenicity of the LNP increased. Introduction of branching in the lipid tail has been known to enhance the transfection efficiency. A ten-fold increase in the transfection of mRNA was observed in LNPs with branched chains when compared to LNPs with unbranched control lipids [23]. Lipids with a multi-tail design provide the desired intrinsic curvature by increasing the cross-section of the tail region. This increase in cross-section is imparted by the presence of more than two tails in the lipid. LNP’s prepared with C12-200, a multi-tail lipid, showed a seven-fold increased potency when compared to a control formulation [24]. The biodegradability of lipids with multi-tails, however, should be carefully optimized to avoid accumulation in body and their subsequent potential toxicity [21]. Polymeric lipids are another type of lipids where the lipid tail is further branched and is more complex than other lipid tail structures explained so far. Polyethyleneimine lipids, e.g., 7C1, have been shown to provide efficient siRNA delivery to non- liver targets [25].

The lipid tail designs discussed here impart necessary endosomal drug release properties to the LNP; however, at the same time, it is critical to retain drug cargo during blood circulation, especially for LNPs that are delivered systemically either intravenously or subcutaneously.

Drug cargo retention for systemically administered LNPs depends on the existence of constituent lipids in either a gel or liquid crystalline phase. This gel and/or liquid crystalline structure is dependent on the melting temperature (Tm) of the lipids. The Tm in turn is governed by the lipid carbon chain length or unsaturation. Shorter or unsaturated lipid chains tend to have lower Tms than longer and saturated chains [26,27,28,29]. At physiological temperature, the low Tms of shorter (e.g., 1,2-dimyristoyl-sn-glycero-3-phosphocholine (DMPC) and 1,2-dipalmitoyl-sn-glycero-3-phosphocholine (DPPC)) and unsaturated (e.g., 1,2-dioleoyl-sn-glycero-3-phosphocholine (DOPC)) lipid chains impart a more fluidic character with loose packing, as the order of packing decreases with the decrease in Tm. Branched lipid chains therefore allow for a lowering of the melting point and therefore increase the fluidic character that causes poor packing of lipids and increases fusogencity. 

**Figure 3:**
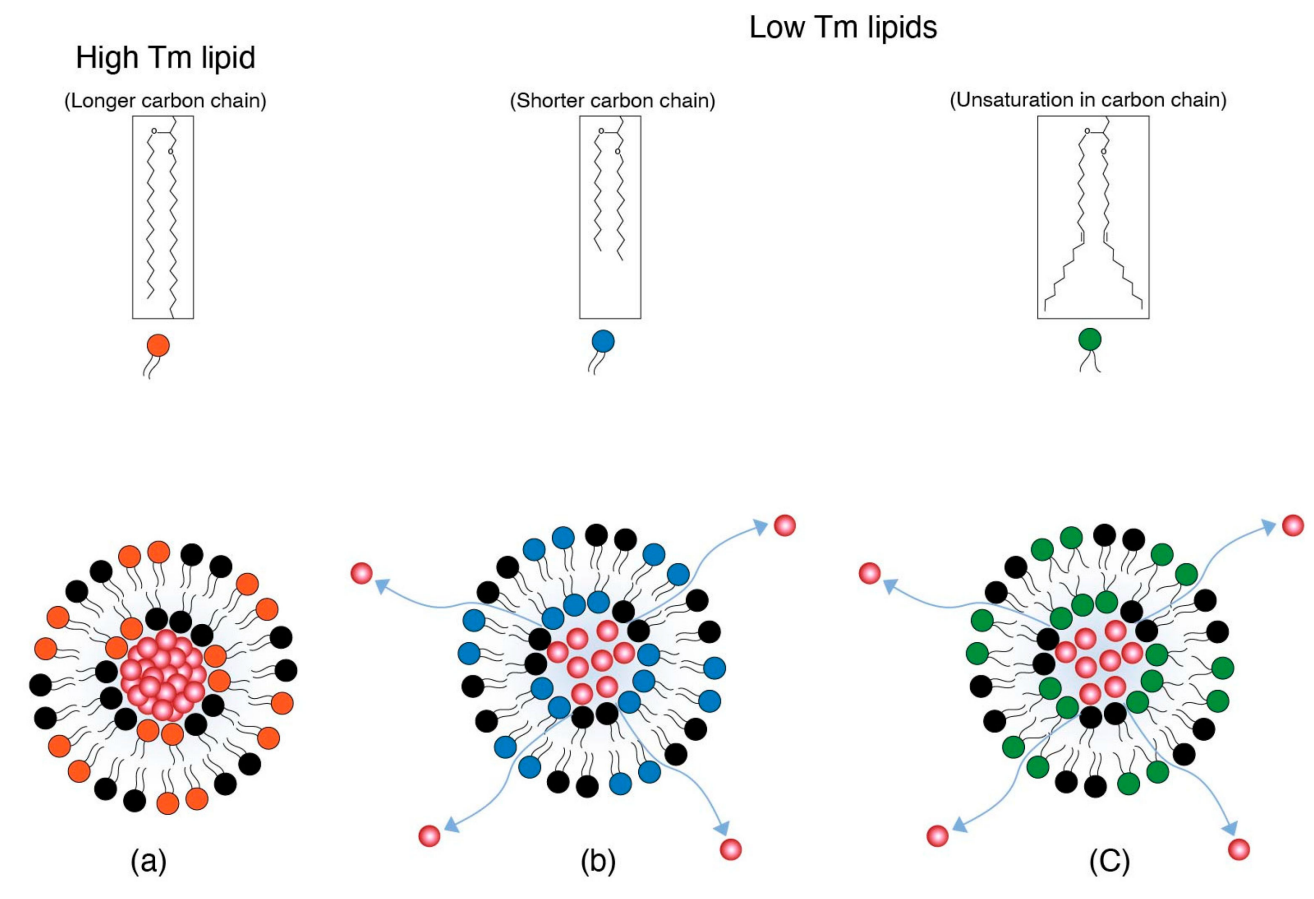
(**a**) An LNP with high Tm lipid represented by red head-group lipid keeps the drug cargo intact. (**b**) An LNP with low Tm lipid with a shorter carbon chain represented by blue head-group lipid and (**c**) An LNP with low Tm lipid with unsaturation represented by green head-group lipid. Both (**b**) and (**c**) impart more fluidic character to the LNP and thereby allow for more leakage of cargo from LNPs.

Depending on the Tm and its similarity to body temperature, the fluidic phase may vary from pockets of the fluid phase to a more homogenous fluid phase of the LNP membranes. Poor packing is a feature of more disorder in the lipid arrangement due to a fluid phase that aids in the destabilization of lipid nanoparticles [30]. The disorder in the membrane packing and therefore membrane disruption also originates from unequal chain lengths of different lipids, i.e., ionizable lipids, helper lipids (e.g., 1,2-dioleoyl-sn-glycero-3-phosphoethanolamine (DOPE)), and PEG-lipids [31]. Disorder in membrane packing is based on the Tm of individual lipids in LNP membranes and leads to leakage of more drug cargo. This is illustrated in Figure 3. The bilayer structure is presented pictorially; however, the concept applies to LNPs as well. 

The fluid nature of lipid membranes is also exploited in mRNA LNP vaccines, where usually short chain PEG lipids, i.e., C14, are used to allow for poor lipid packing in the LNP system and the subsequent rapid disengagement from the LNP. This is described in more detail in the charged-based targeting section in this review. Furthermore, this phenomenon of membrane destabilization has been extensively exploited to prepare thermosensitive liposomes and induce triggered release upon application of heat by phase transition at elevated temperatures [26].

##### Drug Retention within LNPs

While lipid tail designs can be tuned to ensure interaction with endosomes and the release of cargo, drug retention during shelf stability and systemic circulation post-administration is also a critical consideration for drug potency. Drug retention is governed by the carbon chain length of the constituent lipids and their packing in the LNP system irrespective of the drug cargo type, i.e., biomacromolecules or small molecule drugs.

In a study on ionizable lipid design for liposomal formulations by Zheng et al. [32], it was determined in an in vitro study at mildly acidic pH that shorter lipid chains progressively leaked their payload more than longer chain lengths as chain length increased from C10 to C16. This may be due to weaker van der Waals interaction forces at lower chain lengths, leading to content leakage upon acid triggering. This is bolstered by the fact that branched lipid chains without unsaturation and cyclopropyl derivatives of the lipid chain did not additionally trigger content release. The lipid chain length is thus critical in drug retention while an LNP is in systemic circulation.

#### 2.1.2. LNP Surface Charge Measurement/Evaluation

While pH tunability and drug retention are important design elements to consider, accurate measurements of the surface charge is also critical for LNP development. The surface charge is an essential feature of charge-based interactions of LNPs within target cells. It is therefore critical that pKa measurements, that are critical for the tunability of ionizable lipids, are indeed assessed correctly. Some key points in regard to pKa measurements are discussed here.

It should be noted that the surface charge of individual ionizable lipids is also different from that of the LNP composed of these ionizable lipids. This may be due to the change in electron density of the ionizable lipids due to the presence of non-ionizable lipids which may impact their tendency to protonate. This difference may also be the result of the difference in proton solvation energy in the LNP phase vs. the aqueous phase [33]. 

**Figure 4:**
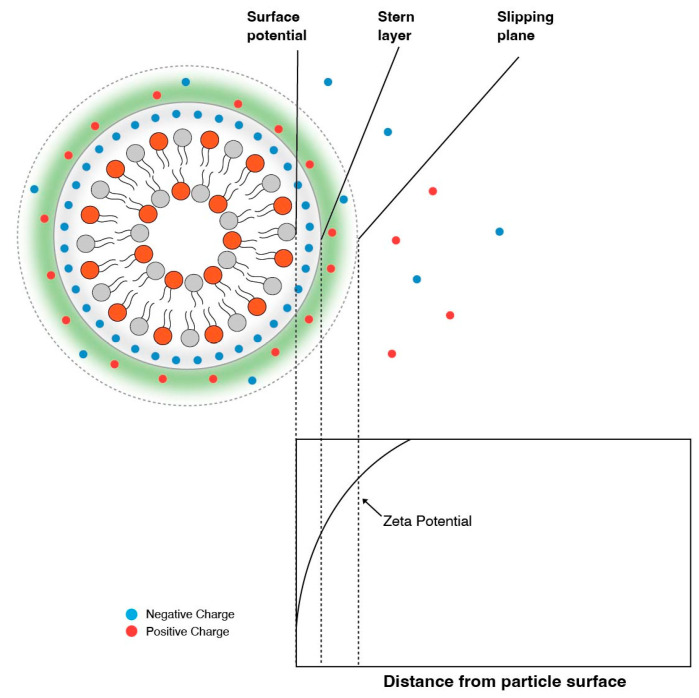
The zeta potential is the potential at the slipping plane and tends to be higher (or lower) than the potential at the surface.

It is therefore essential to experimentally determine the pKa of the final LNP composition. It is important to note that experimentally determining the pKa with techniques such as using 2,6-toludinyl-naphthalenesulfonate (TNS) will be different then when assessed by zeta potential measurements. Zeta potential is the electric potential at the electric double layer, i.e., slipping plane [4,34], and is dictated by the presence and strength of counter ions, as shown in Figure 4. TNS assays, on the other hand, measure the surface potential of the particle. TNS is a negatively charged fluorescent dye that binds to the positively charged surface, resulting in an increase in fluorescence [35]. Fluorescence vs. pH curves generated by TNS assays are then used to predict pKas. 

The titration of an LNP following a zeta potential measurement shows a shift in pKa because of the charge offset by counter ions. Carassco et al. [33] also determined that zeta potential titration curves are much broader compared to TNS curves. It was concluded that this was the result of zeta potentials being lower in general than the surface charge determined by TNS. 

Another important factor that affects the surface charge is the presence of PEG chains with a high concentration of PEG polymer chains that shield the surface charge [36]. This is beneficial during blood circulation [26], as it evades RES and enhances clearance in the body, but it is critical to acknowledge charge shielding by PEG. Surface charge should be therefore measured correctly via TNS or other suitable techniques to assess the lipid pH tunability.

Other indirect methods to evaluate the extent of protonation are hemolytic activity tests, membrane fusion assays, or interactions with model liposomes that have a negative charge. Hemolytic assays use red blood cells (RBC) to test their interaction with ionizable lipids in mildly acidic environments. RBC membranes are very similar in composition to endosome membranes [37]. Both hemolytic and membrane fusion assays work on the principal of electrostatic attractions of protonated positively charged LNPs with negatively charged RBCs or model liposomes. Additionally, in membrane fusion assays, LNPs are incubated with fluorescence-labelled endosomal vesicles and the fusogenicity is assessed by the change in fluorescence over time [38]. These assays are also conducted in mildly acidic pH environments. Lastly, model liposomes that have ~15% anionic lipids are used to mimic negatively charged endosomal membranes and are also used to observe the change in particle size upon interaction with ionized LNPs over time [9].

### 2.2. Biodegradability

To avoid a significant accumulation in the target organs and its associated toxicity, lipids used in LNP systems are biodegradable. Some lipids used in FDA-approved products have shown poor clearance, e.g., MC3 [39]. Hassett et al. [5] demonstrated only a 50% reduction in the Cmax value of MC3 after 24 h of intramuscular administration in a rodent model. They also showed detectable levels in the liver and spleen after a 24 h period. Sabnis et al. [7] also observed an extensive accumulation of MC3 in the liver after 48 h of intravenous administration in rats. Ionizable lipids, therefore, usually have an ester group added to their lipid chains to render them biodegradable. The ester on the lipid is a substrate of esterases in the body that cleaves the molecule into more hydrophilic components, i.e., an acid and an alcohol. These breakdown products are than readily eliminated from the body or can be further metabolized [8]. The strategic location of ester groups on the lipid carbon chain determines the length of metabolite. In general, the shorter the metabolite, the more hydrophilic it is and the lower chance it is retained in the body. In addition, it was also demonstrated that the position of the ester group on the lipid chain also dictates the extent of accumulation in target organs. In a rat study, a high lipid accumulation was observed when the ester group was placed near to the head group nitrogen [8]. Therefore, a fine balance between the level of enzymatic cleavage and the hydrophilicity of the breakdown products can be achieved by carefully choosing the ester position on the lipid chain.

### 2.3. Immunogenicity 

Barring specific desired immune responses activated by mRNA LNP vaccines, there are undesired immune responses triggered by LNP systems that are common to both vaccine and non-vaccine LNP systems. 

Undesired immunogenic responses are largely controlled by the size, surface charge, and aggregation of LNPs [40]. Smaller sizes have been shown to favor immune evasion [41]. Much of the size issues have been resolved by present day manufacturing, where LNP sizes of 100 nm or smaller and a polydispersity index (PDI) of ≤0.25 have been ensured [42]. 

A higher charge tends to trigger RES and thus initiates clearance from the body. The surface charge is controlled by the ionization of the lipids that comprise the LNP system and a PEG coating essentially hides these surface charge by presenting a hydrophilic shell around the LNP. This is covered extensively elsewhere in this review. 

Furthermore, an additional aspect of immunogenicity is CARPA, which is an unwanted immune reaction largely directed to PEGylated lipids in an LNP system [26]. To this effect, Suzuki et al. [43] have shown that the rate of anti-PEG antibody generation depends on the rate of PEG shedding from the LNP. They showed that in a mice model, the fast shedding rates of PEG lipids from the LNP surface have been known to lead to less anti PEG antibodies compared to slow shedding PEG lipids [43].

## 3. LNP Delivery—Intravenous (IV), Intramuscular (IM), or Subcutaneous (SC)

So far, we have discussed the design of LNPs; however, there are some critical considerations for the site of administration of LNPs as well. Intravenous administration is the preferred route of administration for therapeutic indications where the expressed protein needs to be circulated systemically or a deeper tissue penetration is required which is not achieved by the IM or SC routes [44,45]. 

The IM route, on the other hand, is preferred where the intended target is localized skeletal muscle, draining lymph nodes, or resident macrophages. As for IM administered COVID-19 vaccines, the target cells are muscle cells or antigen presenting cells (APCs), such as dendritic cells or monocytes where mRNA translation occurs, and the translated protein (spike protein) is then quickly accessed by B and T cells via the draining lymphatic system [46,47]. Due to this quick access to target cells, the residence time for intramuscularly administered LNPs is shorter than the intravenously administered product. 

Such a direct access to immune (B and T) cells is challenging with subcutaneous tissue administration as subcutaneous tissue can cause slow diffusion of large molecules and therefore a delayed presentation to immune cells [48]. This may result in a loss in efficacy. Di et al. [49] showed poor efficacy when mRNA LNPs were administered via the SC route as compared to IV or IM in a BALB/c mice study.

## 4. LNP Target—Hepatic and Non-Hepatic

### 4.1. Hepatic Targeting 

For IV administered LNPs, the natural target organ is the liver due to ApoE-mediated cell uptake. Hepatic accumulation is, therefore, considered the most significant barrier in advancing mRNA-LNP therapeutics to extrahepatic targets. The main reason for LNP build up in the liver is the presence of large vasculature in liver sinusoids and relatively slower blood flow, which results in extravasation of LNPs in the liver. Hepatic targeting is a two-step process that starts with PEG shedding followed by internalization by hepatocytes and subsequent endosomal escape.

#### 4.1.1. PEG Shedding 

The PEG shedding strategy used in recently developed LNP systems is based on PEG lipid desorption from the LNP surface upon entering systemic circulation. The PEG coating on one hand prevents the aggregation of liposomes upon storage and to some extent in vivo also, but on the other hand hinders interactions of lipid nanoparticles with target cell membranes and their subsequent internalization [50,51]. Ideally, the PEG layer should be removed at the site of action.

The way the PEG coating is exploited for targeting/stability has varied over the years by either making it a more stable component of LNP systems to evade undesired immune responses while in systemic circulation, as in the case of intravenously administered LNPs, or by applying the PEG coating as a transient component which provides stability upon storage but sheds in vivo upon interaction with serum proteins, as is the case for intramuscularly administered LNPs. The shedding of PEG relies on (i) shorter carbon chain lengths of the lipid that is conjugated to the PEG, (ii) the concentration of serum proteins in vivo, and (iii) the time of exposure to serum proteins [52]. In an in vitro study, Gallud et al. [52] demonstrated an increase in the uptake of PEGylated LNPs in Huh 7 cells when LNPs were pre-incubated in fetal bovine serum (FBS). Moreover, the increase in LNP uptake was proportional to the amount of FBS present and the time of incubation. They also showed that LNPs anchored to streptavidin via Biotin-mediated binding were released as the medium was exposed to FBS, providing a clear insight into the FBS-mediated release of PEG lipids. However, the precise mechanism of stimulation by serum proteins to augment the PEG lipid release from the LNPs is still unknown. 

Additionally, there are other mechanisms that play a vital role in PEG lipid removal from nanoparticles. One such force is the cohesive interaction of PEG lipids within nanoparticles with other lipid components. It is important to note that formation of non-polar lipid structures, such as LNPs/Liposomes, depends not only on the hydrophobic interaction of non-polar lipids but also on the van der Waals forces of interaction among lipid tails of constituent lipids [53]. Lipids with shorter carbon chains therefore have a lower binding force with the lipid assembly and have more chances of breaking out of the lipid arrangement [54]. An additional trigger for this lipid release from the nanoparticle system could be the large PEG overhang on shorter carbon chain lipids on the nanoparticle surface which is highly water soluble. Another factor that can be used for PEG shedding is branching or unsaturation in the carbon chains, which limit lipid packing and reduce the melting point and subsequently the stability/lipid desorption in vivo [55]. Whatever the PEG shedding method, once the PEG layer is removed, the exposed LNP surface can freely interact with the target membranes.

#### 4.1.2. ApoE Based Hepatocyte Internalization

**Figure 5:**
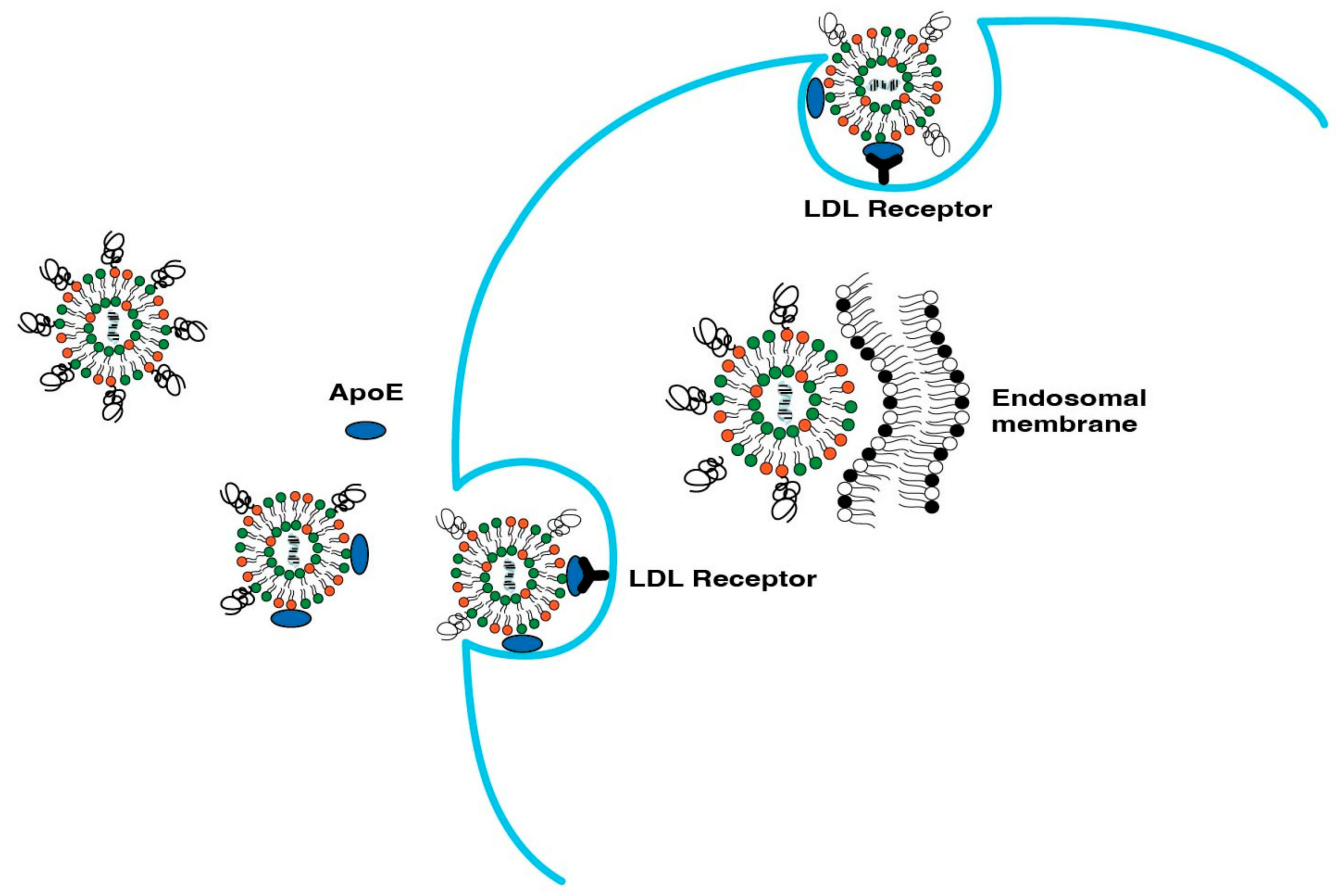
ApoE-mediated uptake of LNPs in hepatocytes.

Hepatocyte internalization of LNPs occurs by two separate mechanisms: (i) ApoE-mediated uptake and (ii) endocytosis. Once in systemic circulation, ApoE binds to LNPs, which leads to their accumulation in hepatocytes and internalization via low density lipoprotein (LDL) receptors in hepatocytes. Figure 5 illustrates the ApoE-mediated hepatic uptake of LNPs. Such ApoE-based uptake has been confirmed by the difference in LNP uptake in ApoE^−/−^ mice [56,57]. Another parallel route for the uptake of LNPs is the clathrin-dependent endocytosis mechanism of LNPs [58]. The endocytosis route has been exploited by interactions based on electrostatic charge that have the potential to enhance cellular uptake. Such charge-based approaches are described subsequently in this article.

### 4.2. Non-Hepatic Targeting 

As in hepatic targets, there are no direct carrier (ApoE) mediated targeting avenues for extra-hepatic targets. Several approaches, therefore, have been employed for non-hepatic targets. In this regard, selective organ targeting (SORT) has been explored by Dillard and co-workers [59], where a fifth component (or SORT molecule), in addition to the conventional four-lipid composition of LNPs, enables extrahepatic targeting of LNPs. The authors hypothesized that after desorption of PEG lipids on the LNP surface, the underlying SORT molecules are exposed and recognized by distinct serum proteins. These serum proteins then adsorb onto the LNP surface and lead the LNPs to the targeted organ by interacting with cognate receptors expressed by cells in the target organs, akin to the ApoE-based LNP accumulation in the liver. The group successfully demonstrated liver, spleen, and lung delivery, and has shown potential for other important delivery targets. The SORT molecule library also consists of lipids of diverse pKas. It was determined that 6–7 pKa lipids are best suited to liver targeting, greater than 9 pKa lipids are ideal for lung targeting, and 2–6 pKa lipids are best suited to spleen targeting [59].

Meyer et al. [60] have also thoroughly reviewed extrahepatic targeting for mRNA delivery, which was found be in concurrence with the SORT approach, where positively charged lipids and negatively charged LNPs were found to preferentially accumulate in the lungs and spleen, respectively. Additionally, poly-beta amino ester (PBAE) polymer [61,62], 1,2-di-O-octadecenyl-3-trimethylammonium propane (DOTMA) lipid, 1,2-dioleoyl-sn-glycero-3-phosphoethanolamine (DOPE) lipid [63], and polyacrylic acid (PAA) polymers [64] were found to target the lungs by altering the PEG lipid, helper lipid, and N/P ratio. Similarly, LNPs based on PEG-PAsp (TEP) (polyaspartate w/side chains of four aminoethyl units) [65] and poly(N-isopropylacrylamide) (PNIPAM) [66] were found to be associated with tumor delivery through the EPR effect.

The inherent properties, composition, pKa, size, and charge modulation of LNPs have been found to have great potential for targeting LNP to different tissues. Further enhancing ligand-based modifications is of great value and has been successfully evaluated all throughout the development history of non-viral delivery vectors [67,68,69].

## 5. Charge-Based Targeting Approaches

Charged-based targeting approaches can be categorized in two ways. The first is endosomal escape and the second is target cell internalization in targets with endogenous triggers, e.g., low pH in the solid tumorinterstitium. 

### 5.1. Endosomal Escape

A well-designed LNP system ensures high accumulation and drug cargo release in the target organ/cells, a better safety profile, and lower immunogenicity. For hepatic and non-hepatic targets alike, once the LNP is internalized by target cells, it is entrapped inside the endosome which then converts to a lysozyme and leads to degradation of the carrier and the payload. To avoid this degradation, LNP surface-charge-mediated endosomal escape comes into play. 

The mechanism of action of charge-mediated drug delivery lies in the fact that body cell membranes are negatively charged. It is estimated that cell membranes contain ~15 mol% of negatively charged lipids, i.e., phosphatidyl serine (PS) and phosphatidyl inositol (PI) lipids, in their membranes [70,71,72]. Moreover, the endosomes, where the LNPs are internalized, are also made of cell membranes and have a similar surface charge to that of the cell membrane [73,74]. The net positive surface charge on the nanoparticle surface interacts with the negatively charged membrane and thereby plays a role in drug payload release.

The surface charge of a lipid nanoparticle is used to target the lungs, macrophages, and the spleen [33,75]. Historically, cationic liposomes have been used to target macrophages in the reticuloendothelial system (RES) to treat infections [76]. Early work in the field focusing on cationic liposomes and drug complexes greatly enhanced drug encapsulation and retention, along with enhancing the cellular uptake and cytosolic delivery through better endosomal escape [77]. This led to a deeper and greater exploration of charged nanoparticles. However, for targets outside of the RES, cationic liposomes pose a challenge in that they are readily picked up by negatively charged serum proteins. Such binding is called opsonization and triggers further recognition and elimination by the RES system. In addition, cationic liposomes induce toxicity by activating the complement system and triggering an immune response that may cause inflammation and tissue damage [78]. As an alternative to cationic liposomes, anionic liposomes show a better stability and safety profile and have been used to target organs outside of the RES, e.g., lung cancer [79]. However, since the cell surface is a negatively charged phospholipid bilayer membrane with embedded biomolecules in a fluid mosaic structure [80], cell membranes pose a challenge for anionic NPs, as they are repelled by the cell surface leading to the cargo being unable to reach the site of action. Additionally, the key challenge with anionic liposomes is the poor encapsulation of genetic material [81]. 

Since the surface charge of LNPs can lead to rapid clearance by the RES, the concept of ionization at the target site comes into play. All these factors collectively reduce the therapeutic potential provided by charged NPs and that is where ionizable NPs come into play. Ionizable NPs remain neutral at a physiological pH, circumventing the limitations posed by charged NPs. LNPs fill the gap perfectly by offering inclusion of ionizable lipids for efficient cellular uptake, enhancing endosomal escape by gaining a positive change in the endosomal microenvironment and offering a longer circulation half-life, as provided by readily insertable PEG–lipid conjugates for circumventing undesirable phagocytic uptake. The ionization of LNPs is achieved by employing either ionizable lipids or pH-sensitive lipids, as explained subsequently in this review. 

#### 5.1.1. Endosomal Escape Via LNPs with Ionizable Lipids

Since the net charge on the LNP surface is known to trigger the RES and thus cause their rapid blood clearance, ionizable cationic lipids, such as DLin-MC3-DMA, ALC-0315, and SM-102, have been designed such that they remain neutral at a physiological pH and acquire a positive charge by protonating in a low pH endosomal environment to assist in endosomal escape of the payload. Upon endocytosis of the delivery system, the pH in the endosomal vesicles further drops to 5.5–6 in the endosome and 4.5–5 in the lysosome [82]. As explained earlier in this review, an acidic environment triggers protonation of the ionizable lipid constituent of the LNP, thereby enhancing the surface charge of the LNP and the subsequent release of drug cargo.

An efficient LNP design ensures not only successful internalization by target cells but also by the efficient and timely release of the contents inside the target cells. All LNP-based therapeutics, shown in Table 2, are prime examples of this strategy. 

#### 5.1.2. Endosomal Escape Via LNPs/Liposomes with pH-Sensitive Lipids 

pH-sensitive lipids have been used largely with pH-sensitive liposomal systems over LNPs. pH-sensitive lipids are lipids with linkers that can cleave or undergo conformational changes at lower pHs and are typically employed for endosomal escape. Such lipids with pH-sensitive linkers are more stable in systemic circulation but cleave in low pH endosomal environments, and are therefore used to prepare such pH-sensitive liposomes. Moreover, such systems are presumed to have a good shelf stability [83] owing to their stability at physiological pH. Here, we describe commonly used linkers that aid in endosomal escape in a manner similar to LNP interaction with the endosomal membrane. Additionally, pH-sensitive PEG lipids can cleave at low pHs to dePEGylate the LNP membrane. pH-sensitive lipids are also used to dePEGylate by protonation which leads to the PEG chains concentrating in one domain of the LNP surface, enhancing the interaction with the endosomal membrane [9]. 

Lipids with pH-sensitive linkers undergo acidic hydrolysis in the endosomal environment, which aids in drug release either by disrupting the LNP membrane or by increasing the interactions with the negatively charged endosome membrane. Commonly used acid labile linkers are acetals, ketals, vinyl ethers, and orthoesters. There are other linkers, e.g., trans-2-aminocylohexenol (TACH), that instead of cleaving the lipid, change conformation under acidic conditions and enable drug release. pH-sensitive linkers commonly used in charge-based targeting are discussed here. A list of such pH-sensitive linkers is provided in Table 3.

##### Trans-2-aminocylohexenol (TACH)

**Figure 6:**
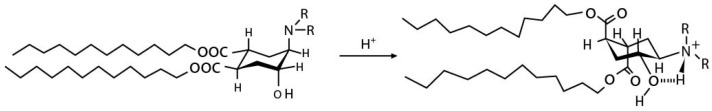
Conformational switch of trans-2-aminohexanol [84].

**Figure 7:**
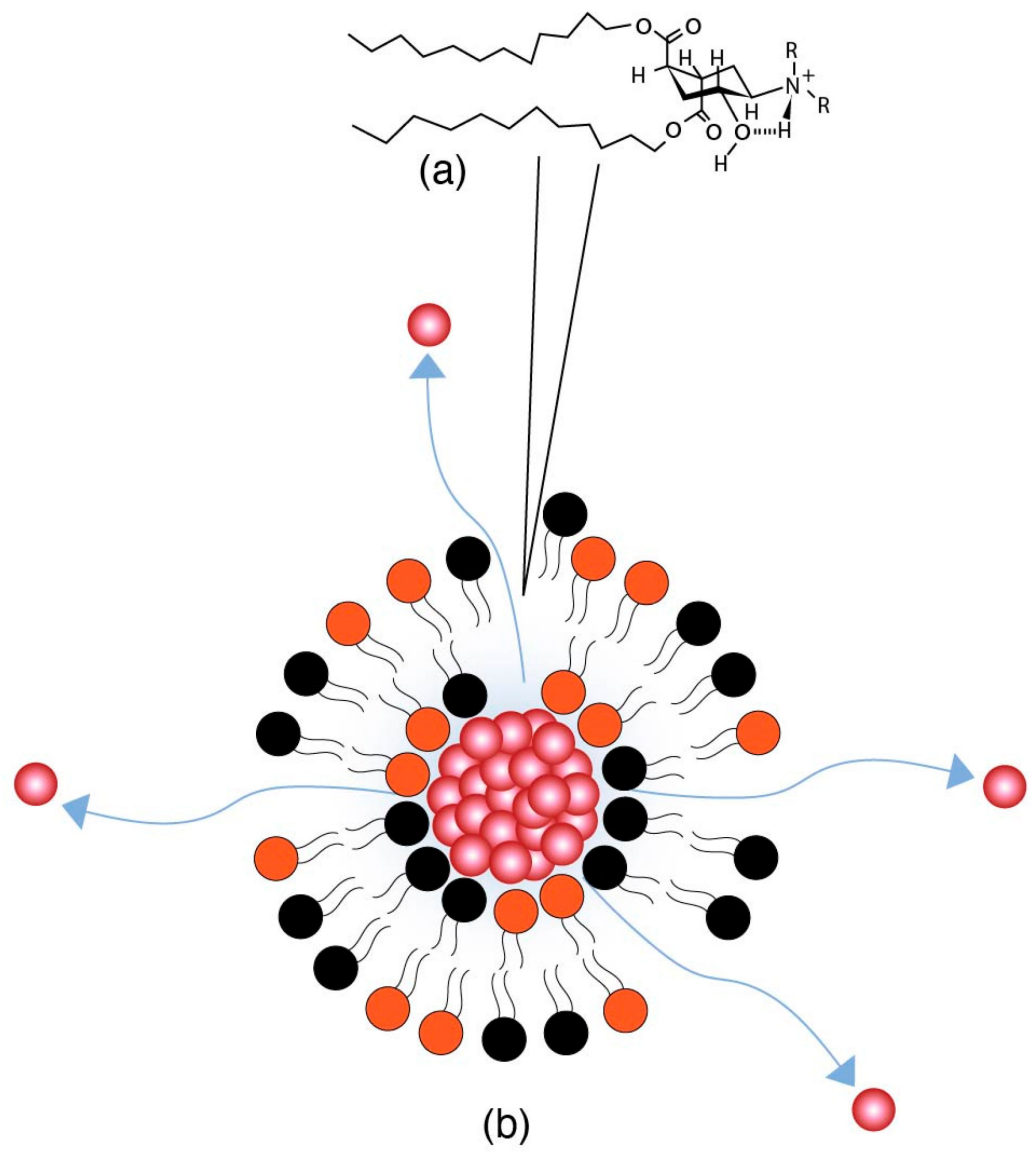
(**a**) Conformational switch of TACH in a low pH endosomal environment. (**b**) Release of drug contents from the Fliposome after the conformational switch of the TACH lipid on the Fliposome surface.

Trans-2-cyclohexanol (TACH) is relative new pH-sensitive linker used for intracellular drug delivery (Table 3). This linker acts by a confirmational switch of TACH under the acidic conditions of the endosome that essentially disrupt the liposomal membrane to release the drug contents (Figure 6). Liposomes made out of these lipids are termed as “Fliposomes” owing to the confirmational switch of the lipids [84]. Figure 7 provides a schematic of the conformational switch and drug release. Zheng et al. [32,84] demonstrated that the pH sensitivity depends on the polarity of the lipid headgroup and the shape and fluidity of the lipid tails. The basicity of the lipid headgroup provided the necessary pH trigger which was shown by release of an encapsulated fluorophore. A library of TACH-based lipids have been synthesized and fliposomes were prepared with POPC and PEG-ceramide.

##### Acetal Group

**Scheme 1:**
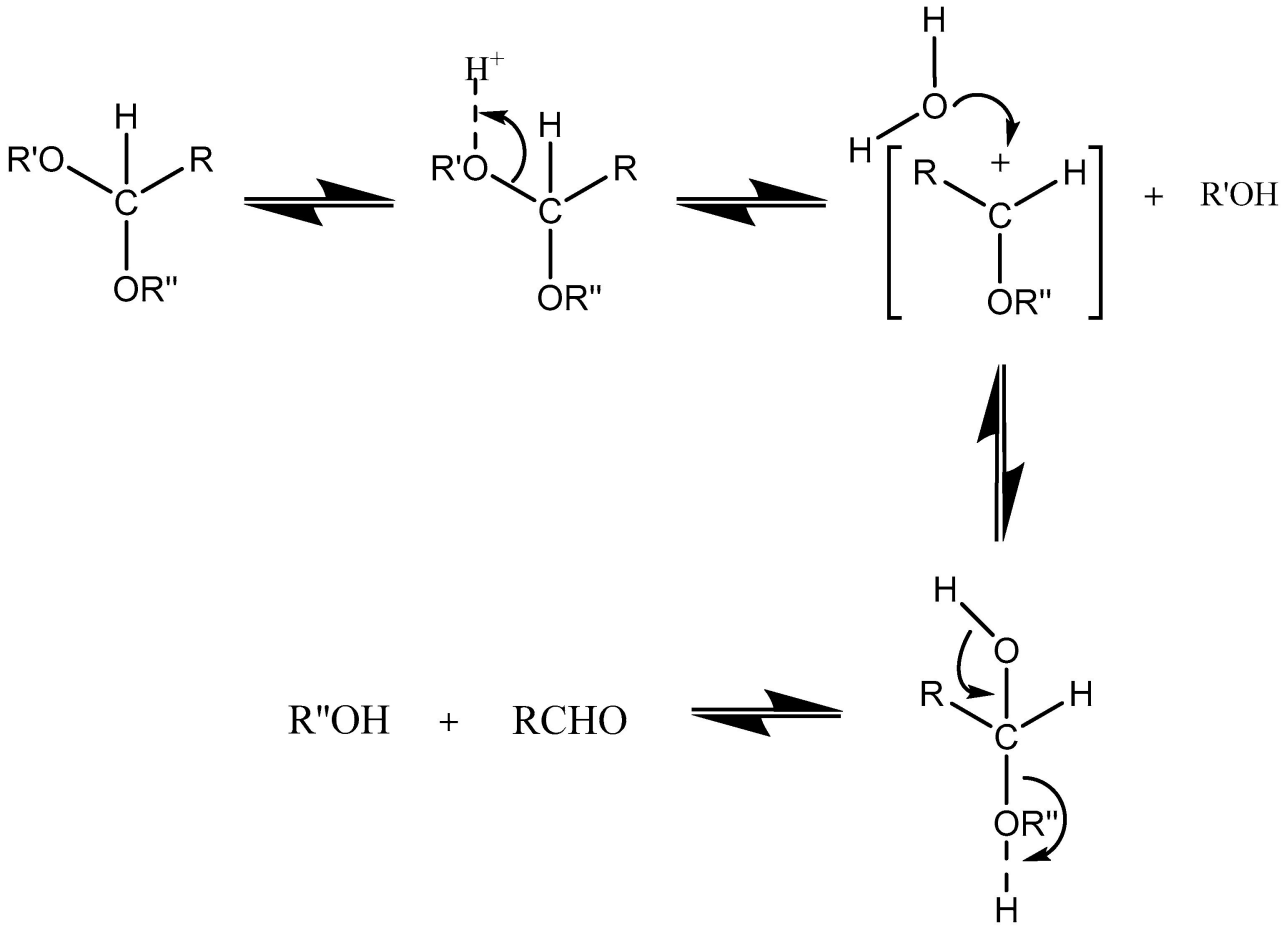
An acetal group degrades to an alcohol and an aldehyde group upon acidic hydrolysis [86].

Acetals are formed by a condensation reaction of alcohols with aldehydes, creating alkoxy groups in the molecule. An acetal group (Table 3) acts as a pH-sensitive linker by undergoing acidic hydrolysis as shown in the Scheme 1 [85,86,87,88]. The linker essentially degrades to an aldehyde and an alcohol upon hydrolysis.

**Figure 8:**
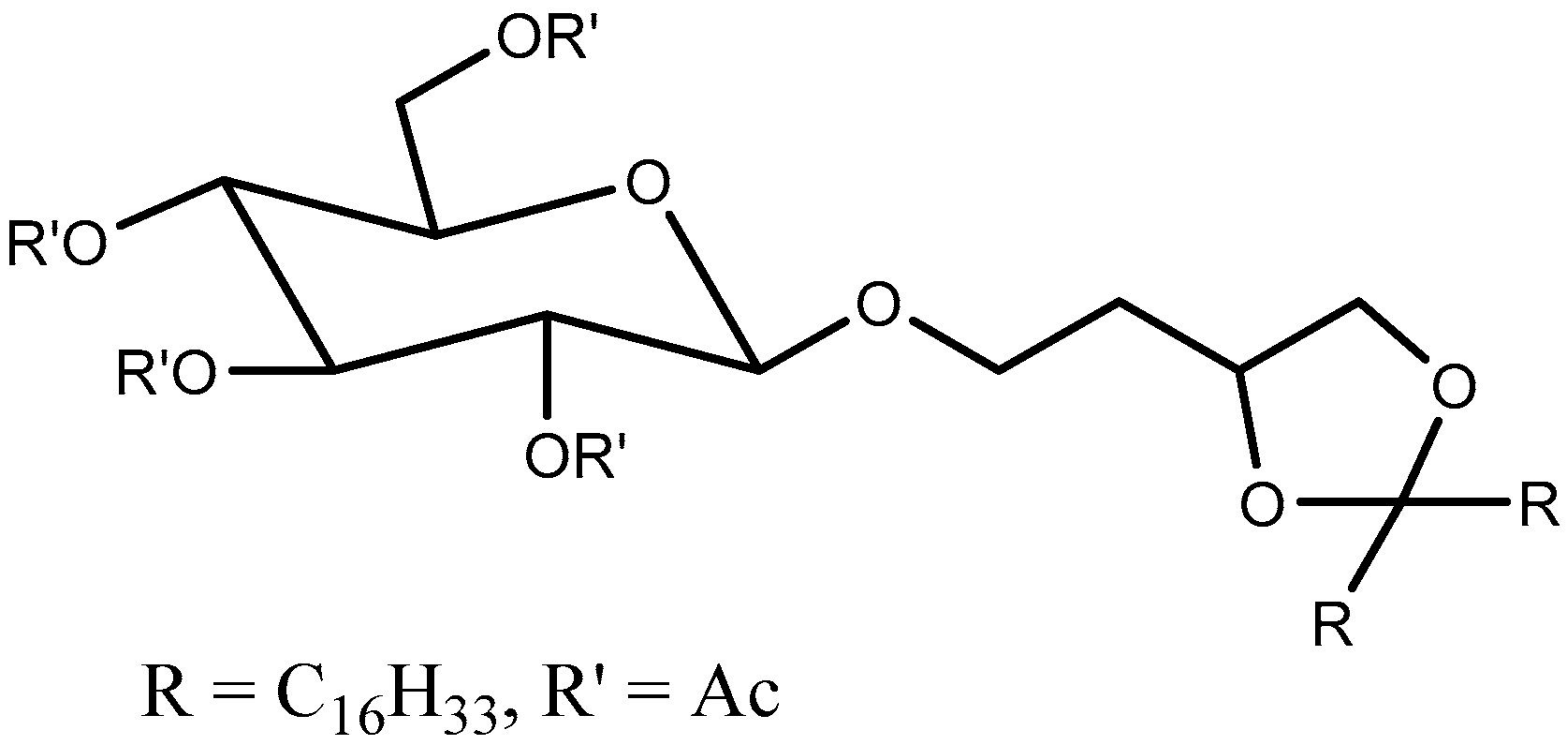
pH-sensitive acetal-based glycolipid [90].

Gillies et al. [87] prepared a series of drug conjugates with polyethylene oxides (PEO) using acetal linkers and were able to tune the pH-sensitivity to attain half-lives of <1 min. to several days. Similarly, Zhong et al. [89] prepared polymeric micelles that provided a good stability at pH 7.4 and pH-triggered doxorubicin release in cancer cells upon endocytosis. Song and Hollingsworth [90] designed pH sensitive glycolipids that were acetal-derived (Figure 8). The pH was measured in ethanol and they demonstrated cleavage upon addition of deuterated HCl, monitored using NMR, while addition of acetic acid (mildly acidic) did not show any cleavage within 14 h. The pH sensitivity was, however, not tested in vivo.

In another study with polymeric systems, drug–polymer conjugates using acetal linkers showed an increased drug release in an intra-tumoral environment. The in vitro studies confirmed a pH-dependent paclitaxel release from the polymer–drug conjugates [91,92,93].

**Figure 9:**
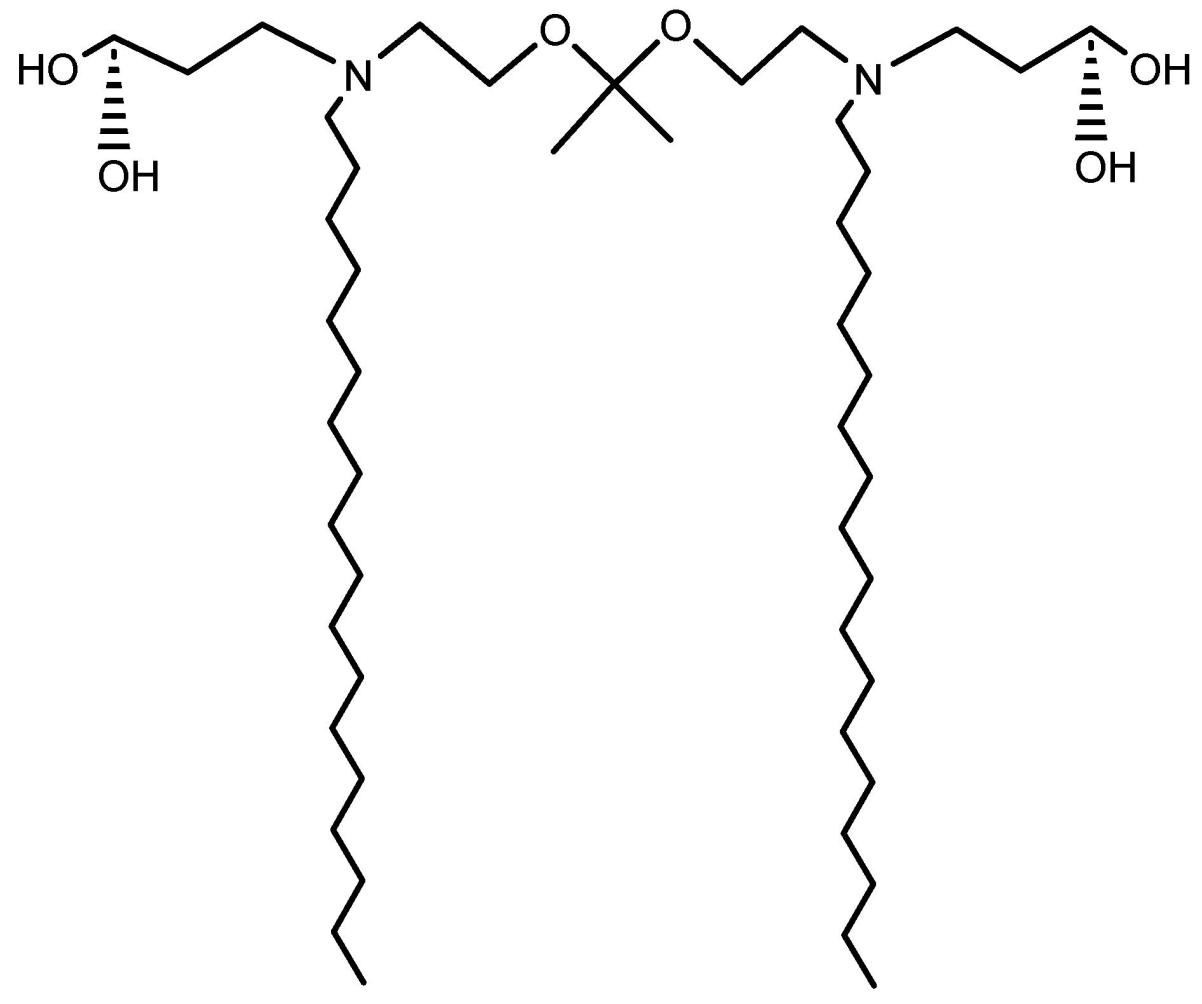
Acetal-based BD2 lipid [94].

**Scheme 2:**
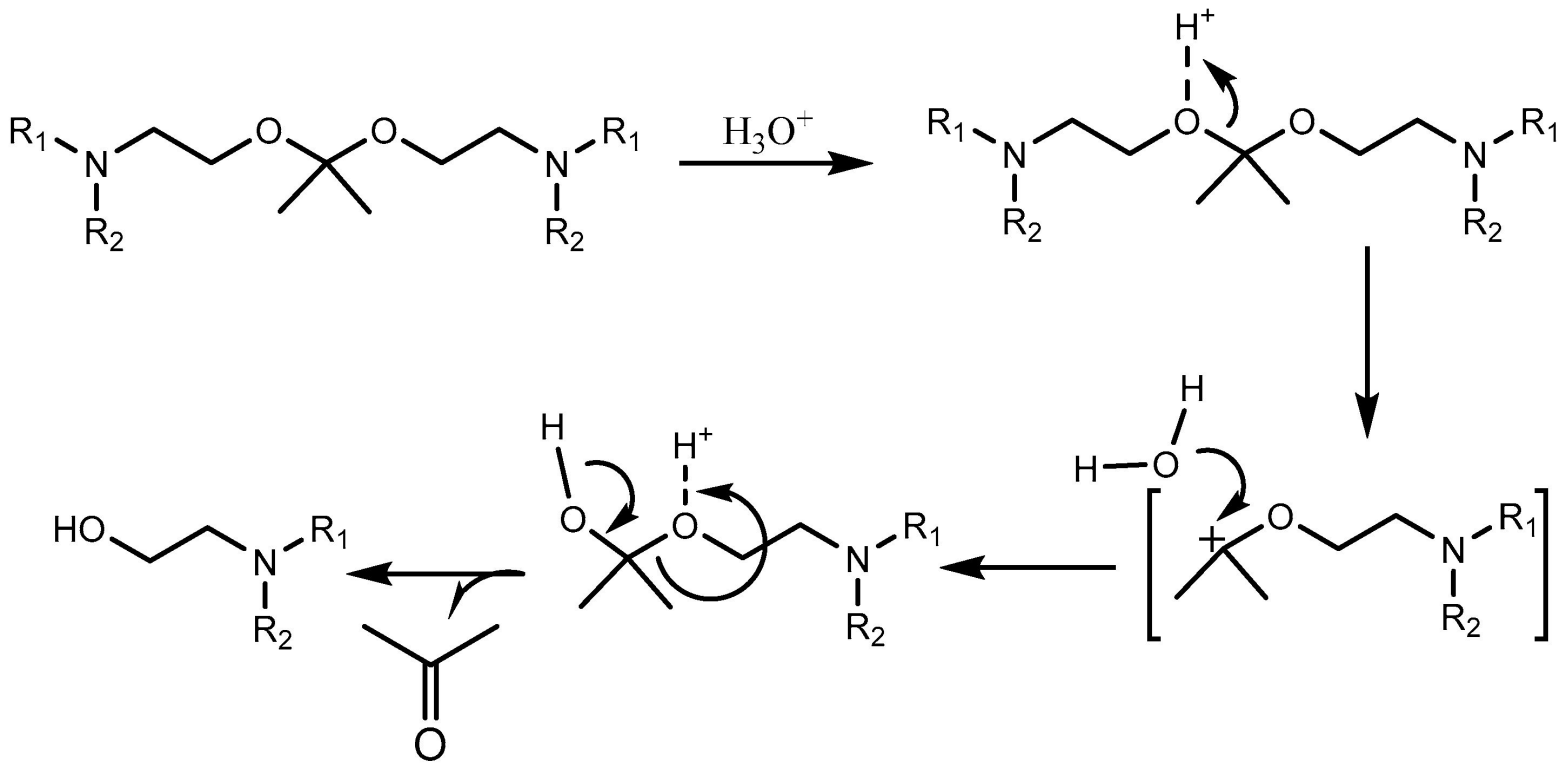
Acidic hydrolysis of BD2 lipid shows degradation into individual lipid entities [94].

Asokan et al. prepared liposomes [94] with a “bis-detergent” BD2 (Figure 9) constituent lipid that was synthesized by using two tertiary amine lipids via an acetal linker. Liposomes prepared by such lipids showed complete hydrolysis at lysosomal pH and a very short half-life of 3 h at endosomal pH, confirming the efficacy of the delivery system in releasing drug contents. They also demonstrated the delivery of oligonucleotides using these BD2 lipids. The release was mediated by disruption of the liposomal membrane integrity by the cleavage of BD2 into separate lipid entities (Scheme 2).

##### Vinyl Ether Group

**Scheme 3:**
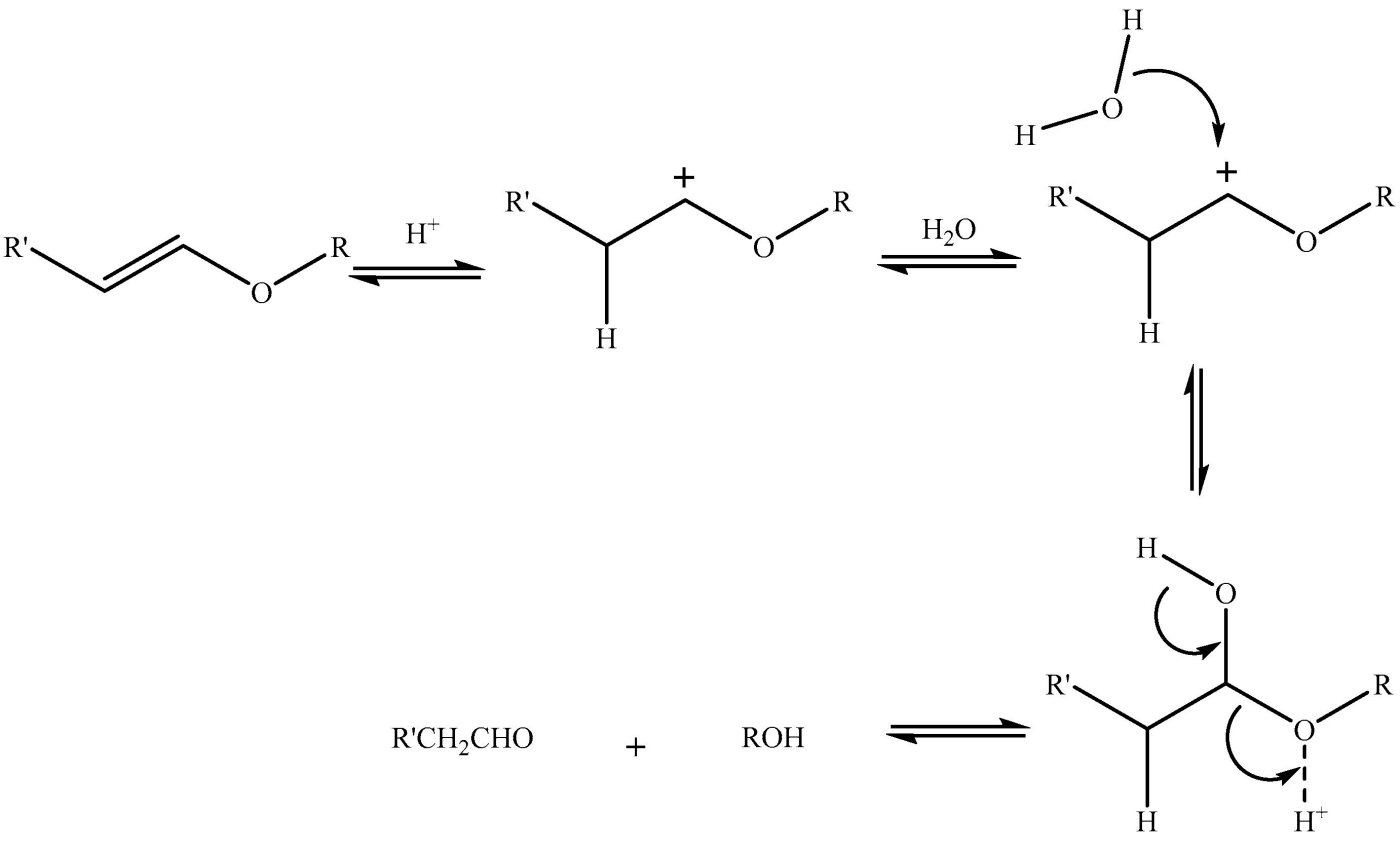
Acidic hydrolysis of vinyl ether [96].

Another pH-sensitive linker that shows in vitro stability at physiological pH but undergoes hydrolysis in an acidic environment is the vinyl ether [95,96,97,98] (Table 3) linker (Scheme 3). Vinyl ether is more distinct from other linkers in that it is very stable at physiological pH compared to other linkers [99]. Acidic hydrolysis leads to the formation of an aldehyde and an alcohol as degradant products.

**Figure 10:**
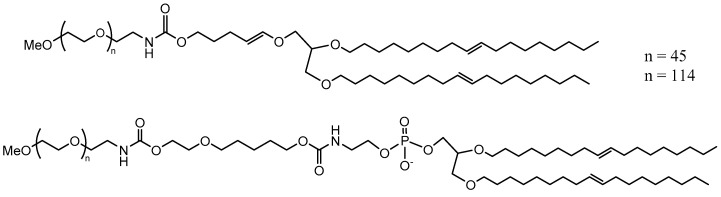
Vinyl-ether-based lipids tested by Thompson and co-workers [99].

Thompson et al. [99] designed vinyl-ether-based lipids (Figure 10) and subsequently a series of DOPE-based liposomes. They demonstrated the acid sensitivity of these systems at pH > 5. The higher ratio of DOPE (>90%) in liposomes ensured the transition from the lamellar to the hexagonal phase. The conversion of the liposome from the lamellar to the hexagonal phase was also dependent upon the kinetics of acidic hydrolysis of the vinyl ether group. The liposomal release profile of calcein dye with 95% DOPE liposome showed significant release within 2 days at pH 4.5. The relatively slower release, on the contrary, provides a better shelf stability at physiological pH. Systems that are less pH sensitive are not suitable for delivery in a tumor environment, where the pH is only mildly acidic (6.5–7.0), but are more effective intracellularly.

In another attempt, Thompson et al. [99] prepared a vinyl ether lipid where they inserted a vinyl ether linker to each of the lipid tails, which then cleaved into two separate lipid molecules, yielding an acid and an aldehyde compound.

**Scheme 4:**
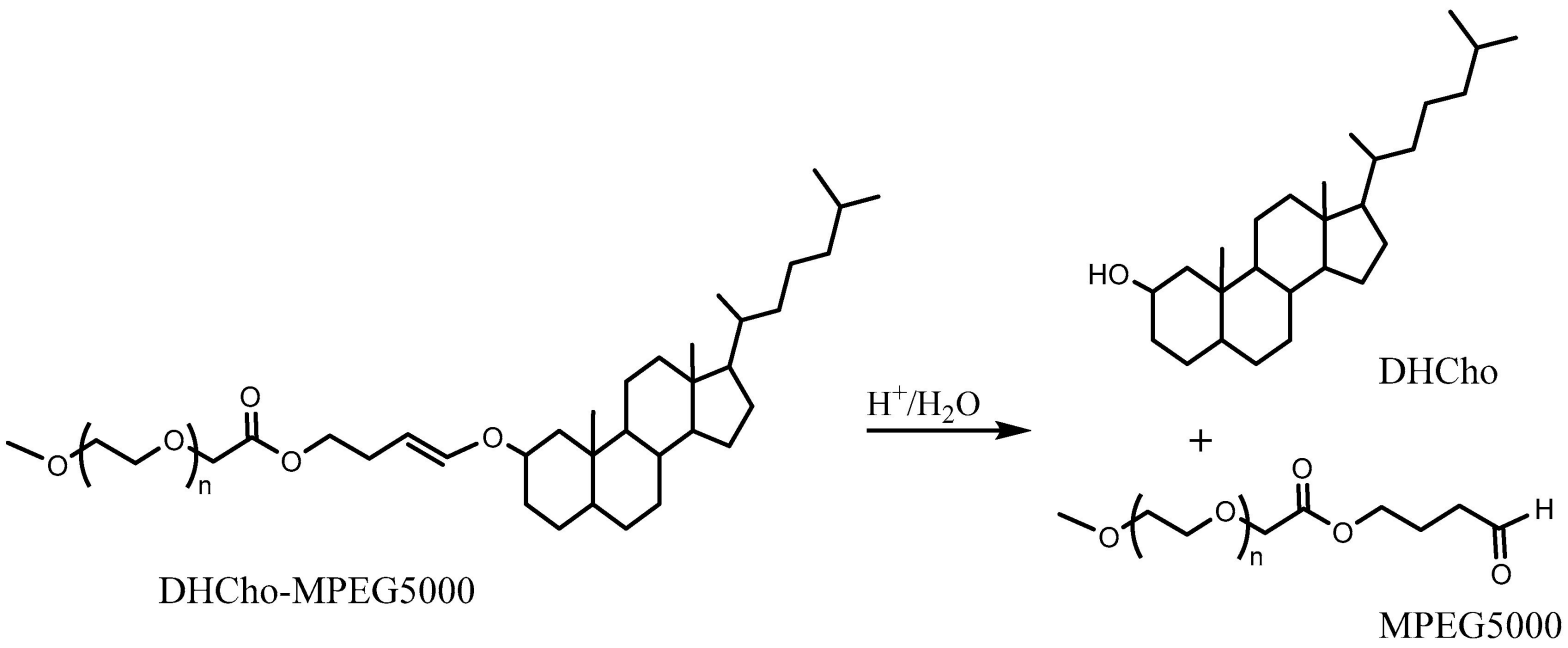
Acidic hydrolysis of DHCho-mPEG5000 into DHCho and mPEG5000 [100].

The release from liposomes prepared with such lipids yielded a faster release of the encapsulated dye. It was shown that it took ~30 h to release more than half of drug cargo at pH ~ 6. The equivalent time to release at ~5 pH was 4 h. A superior pH sensitivity was therefore established by employing two vinyl ether linkers per lipid. In another approach, PEG cholesterol conjugates (DHCho-mPEG5000) were prepared with vinyl ether [100] (Scheme 4). However, the encapsulated content release was limited to ~20% with this approach and therefore did not yield any improvement in pH sensitivity.

##### Orthoester Group

**Scheme 5:**
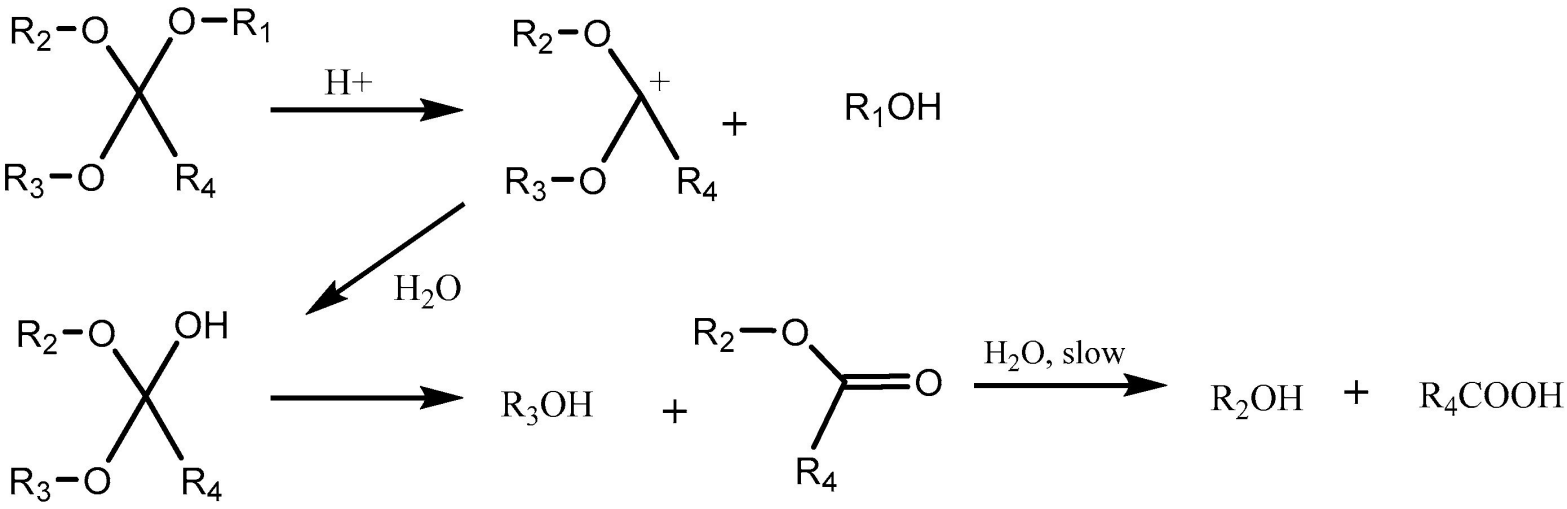
Acidic hydrolysis of orthoesters leading to the formation of an acid and an alcohol [103].

Orthoesters (Table 3) find a place not only as pH-labile linkers in lipid design for liposomal delivery but also as pH-sensitive polymers [101,102]. Orthoesters are more sensitive to pH compared to vinyl ethers but are still relatively stable at a physiological pH. Orthoester-based lipids degrade to an ester compound and an alcohol compound. The degradation mechanism is depicted in Scheme 5.

Szoka and co-workers [103] prepared diorthoester-based “POD” lipids and incorporated them into liposomes. 

POD liposomes prepared with DOPE with a 1/9 ratio showed a lamellar to hexagonal phase transition under acidic conditions. Such liposomes also showed a circulation half-life in mice of ~3 h; on the contrary, for the control liposomes, DSPE-PEG/DOPE, the circulation half-life was ~5 h [103]. The POD-based liposomes showed considerable content release at a pH of 5 [104]. The content release profile was biphasic with an initial lag phase of slow release followed by faster release as the lamellar to hexagonal transition took place. 

POD-based lipoplexes containing plasmid DNA showed rapid release within 2 h at pH ~ 5, while the control remained stable at lower pHs.

In an alternate approach, cholesterol-based lipids or lipids based on straight carbon chain orthoesters were designed and lipoplexes were prepared from such lipids [105]. Stability studies of longer durations were not performed, although the conjugates were stable for several days at a physiological pH.

Besides liposomal systems, the efficacy of orthoester systems has also been established in pH-sensitive block copolymers. Thambi et al. [106] demonstrated DOX release from such pH-sensitive polymers using squamous cell carcinoma (SCC) and cancer cell lines [107].

##### Hydrazone Group

Hydrazone linkers (Table 3) are useful tools under mildly acidic conditions and can also be used for endosomal escape under more acidic conditions.

In contrast to conventional straight chain lipid tails, a library of guanidinium-based lipids with a hydrazone group have shown the desired acid sensitivity [108]. It was demonstrated that unsaturation within the guanidium moiety showed a slower release than when compared to saturated moieties. Lipoplexes made from these systems showed efficient gene delivery in both murine and mammalian cells.

Furthermore, Torchilin [109,110] and co-workers performed extensive work on hydrazone lipids, preparing a series of hydrazone-based lipids where the linker was flanked with either aliphatic or aromatic groups. They showed that by varying the spacers that were attached to the hydrazone linker, the stability at a physiological pH increased. Additionally, the stability of a potential drug product could therefore be ensured. Some of the conjugates showed half-lives of up to 150 min.

### 5.2. Target Cell Interanalization—Solid Tumors

So far, as we have covered charged-based strategies to escape endosome entrapment. However, there are other charged-based target cell internalization strategies where the low pH of the target cell’s surroundings can be used create surface charges on LNPs. An apt example of such an approach is solid tumor targeting. 

Tumor targeting provides an important avenue for charge-based targeting. What makes tumor targeting more unique is that a low pH environment is not only present in the endosome but also in the extracellular tumor microenvironment. The surface charge also helps in tumor targeting, where the pH of the extracellular medium is low, which triggers ionization of ionizable lipids and subsequently increases the interaction with the negatively charged extracellular matrix and cell surface. An efficient targeting strategy ensures removal of the PEG coating followed by endosomal escape, as discussed earlier.

**Figure 11:**
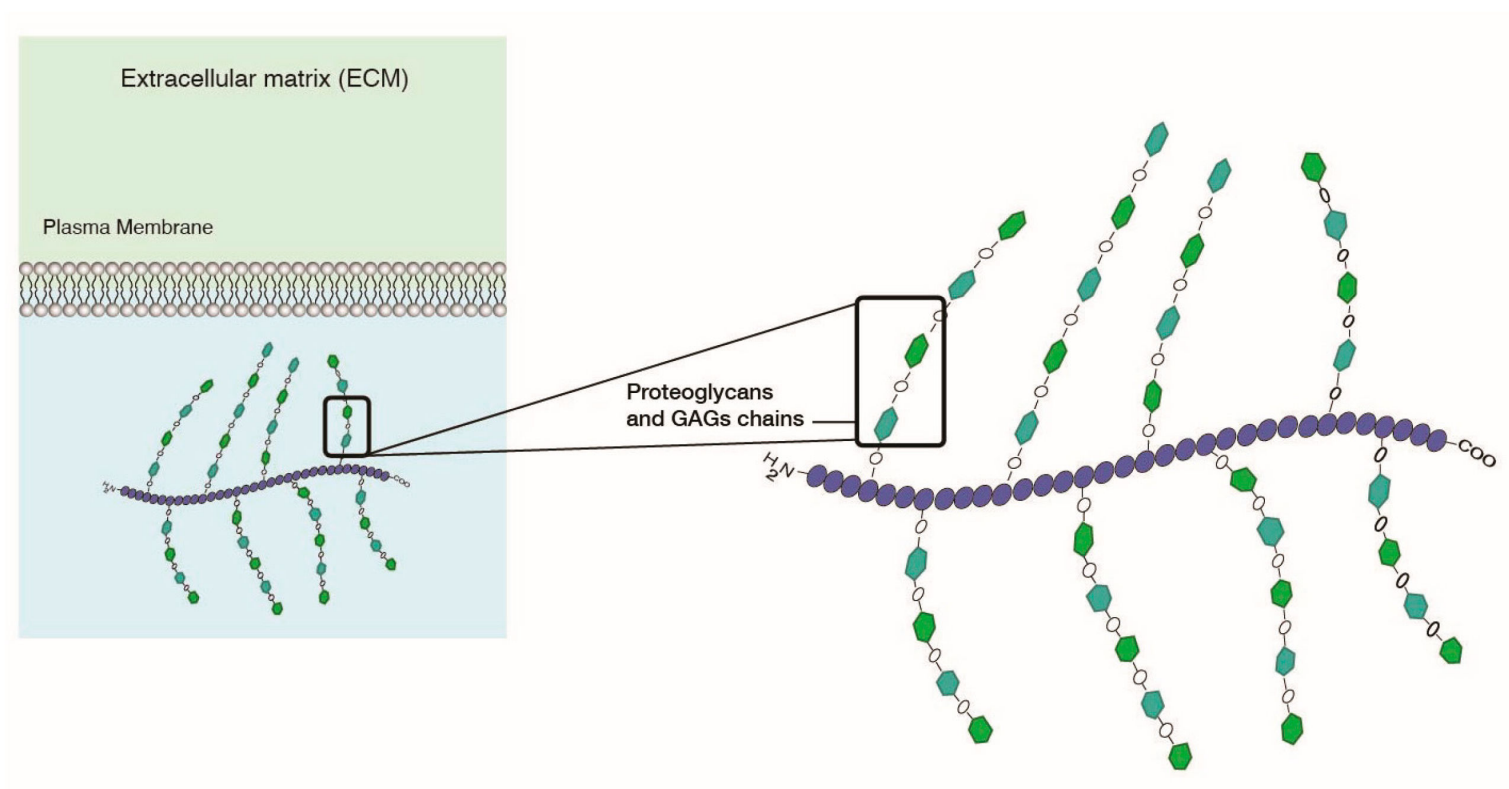
Extracellular matrix has a negative charge due to presence of proteoglycans and GAGs.

Charged-based tumor targeting is based on electrostatic interactions. As described previously, not only do cell membranes have a negative charge, but also so does the extracellular matrix (ECM), due to abundant network of proteoglycans and glucosaminoglycans (GAGs) providing structure to the ECM (Figure 11). GAGs specifically are comprised of hyaluranans, heparins, and chondroitins. The low pH combined with the presence of a negatively charged ECM provides a good targeting lever to develop electrostatic charge-based targeting of LNPs to target cells. Cationic liposomes have been envisioned as a desirable delivery system that can target GAGs [111,112].

Charged-based removal of the PEG coating for tumor targeting largely uses two key approaches: (i) PEG removal by employing a pH-sensitive linker in a PEG-lipid and (ii) concentration of PEG in domains on the LNP surface, thereby leaving a large part of LNPs exposed to target cell surfaces. The incorporation of pH-sensitive linkers in liposomes provides such de-PEGylation strategies. 

#### 5.2.1. PEG Shedding Using pH-Sensitive Linkers

**Figure 12:**
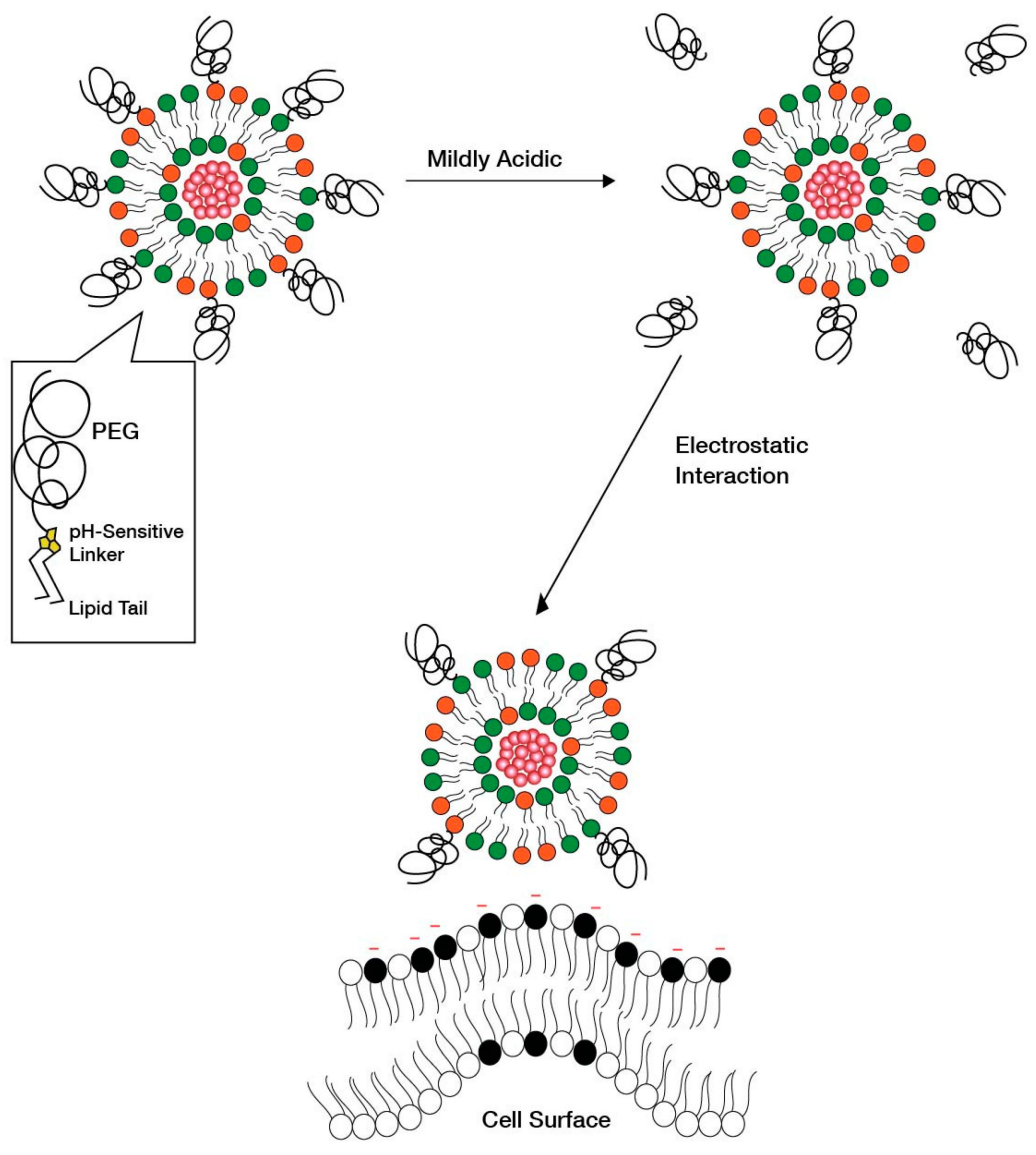
Enhanced interaction of LNPs with the endosomal membrane after dePEGylation via cleavage of pH-sensitive lipids.

Based on this approach, Kanamala et al. [113] showed that pH-sensitive liposomes with hydrazone-based pH cleavable PEG-lipids can shed PEG in the ECM under mildly acidic conditions and can therefore increase the intracellular uptake of LNPs [114]. In another study, hydrazone-based pH cleavable lipids were used to sustain the blood circulation and reduce the clearance of liposomes [115]. A similar strategy was deployed by Zhao et al. [116], where peptides were delivered following cleavage at the acidic site by imine-based pH-sensitive liposomes. Torchilin et al. [109] have also worked extensively in this field by developing a series of hydrazone-based liposomes that can cleave PEG to increase the cellular uptake. Once the PEG coating has been shed in the tumor area, the exposed nanoparticle surface faces tumor cells for enhanced uptake. Additionally, the exposed nanoparticle surface may acquire additional positive charges due to PEG cleavage and have more interaction with the cell surface (Figure 12). Overall, this strategy requires the incorporated pH-sensitive lipid to be cleavable under mildly acidic conditions, i.e., 6.5–6.8, and to avoid premature cleavage, which therefore enhances clearance during systemic circulation. It is also important to ensure that PEG shedding does not destabilize the liposomes and the drug cargo is kept intact during circulation. 

#### 5.2.2. Creation of PEG-Concentrated Domains Using pH-Sensitive Linkers

**Figure 13:**
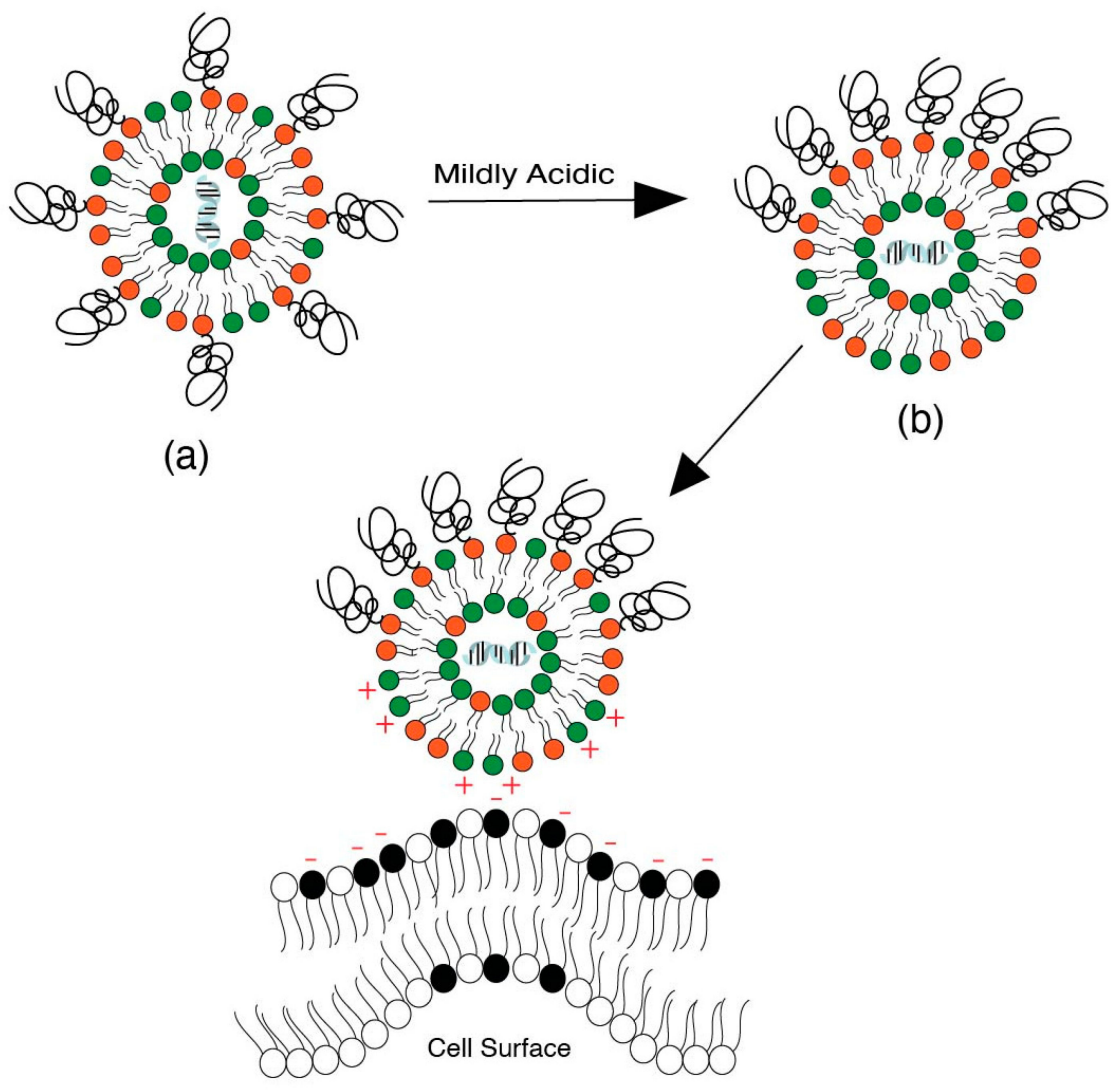
Convertible liposomes that show phase separation of lipids on Liposomal surface [9]. (**a**) Liposomes with uniformly distributed PEGylated lipids on the liposome surface at physiological pH. (**b**) Phase separation of lipids, which leads to formation of domains with a high concentration of PEG lipids on the liposome surface that in turn leads to increased interactions with the negatively charged cell surface.

Another strategy as an alternative to PEG shedding is the “convertible liposome”, where lateral movement of PEG-lipids on the liposome surface allows for an accumulation of PEG in one domain of the liposome [9]. This strategy causes PEG de-shielding, allowing for the LNP surface charge to be exposed and interact with the negatively charged cell surface. Huang et al. [9] demonstrated the lateral movement of lipids on a liposome surface under mildly acidic conditions in a tumor model, which led to an increased uptake of liposomes by revealing positive charges on the surface. Specifically, protonatable imidazole-based lipids were used to form “convertible liposomes”. Such lipids protonate in a low pH environment and acquire a positive charge. These positively charged lipids then interact with negatively charged PEG lipids to give clusters of PEG coating on the liposome surface due to electrostatic interactions between the newly ionized imidazole lipids and the negatively charged PEGylated lipid (Figure 13). Cytotoxic and uptake studies in human and murine cancer cell lines revealed an increase in cytotoxicity and cellular uptake of liposomes with constituent imidazole lipids of increasing pKa. The impact of pKa on the surface charge when in blood circulation and in binding with plasma proteins was, however, not assessed. 

The phase separation of lipids is based on the concept of the formation of lipid rafts on the liposomal surface [117]. Such phase-separated domains can concentrate fusogenic lipids on the liposome surface and increase the process of endocytosis [118]. The phase separation concept is derived from the concept of “lipid rafts” on cell surfaces that are targets for endocytosis of LNPs [119,120].

## 6. Conclusions

The tremendous success of COVID-19 vaccines could have not been possible without LNP systems. Not only mRNA vaccines, but also siRNA-based treatment of polyneuropathy has been approved, as an example of another LNP application. The ongoing clinical trials listed in this article emphasize the breadth of drug cargo that can be delivered through LNPs, as well as the variety of clinical applications that are targeted. From small molecules to ASO, siRNA, mRNA, plasmid DNA, and gene editing, LNPs are being extensively explored for potentially all types of drug cargo. Such medical interventions were thought to be difficult to implement owing to the lack of suitable delivery mechanisms; however, LNPs have provided a big boost to the investigation of all gene therapies, paving the way for the application of precision/personalized medicine much sooner than expected. Many difficult-to-treat health conditions such as oncology, protein replacement, gene silencing, and chronic diseases are now slowly becoming more easily treatable with the advances in this field. Efficient tissue targeting will add more fuel and rigor to the advancements and even gene therapies accessible by ex vivo modulating cells could potentially be replaced by targeting-enabled LNPs and their corresponding nano-delivery systems. It may also prove to be a very handy platform for image diagnostics applications.

Considering the enormous potential that LNPs carry, in this review, we have discussed the approaches for designing lipid nanoparticles that render them more efficacious. We have covered strategies that have been used to design ionizable lipid and helper lipid constituents of LNP systems. Specifically, the ionizable head group design, pKa tunability, and ionizable and helper lipid tail design and concepts were discussed with examples. Moreover, we also covered approaches pertaining to the safety of such systems, such as biodegradability and immunogenicity. We also covered the key considerations and rationale behind selecting the route of administration and targeting of hepatic and non-hepatic targets. We believe that this paper will provide pertinent efficacy and safety-related information for developing an efficient LNP system. 

Moreover, we also discussed charge-based targeting approaches of LNP systems with regard to endosomal escape, and discussed similar strategies employed for target cell internalization in tumor targeting. Charge-based targeting of pH-sensitive liposomes presented in this review should be further explored to advance endosomal escape mechanisms of ionizable lipids and further improve the targeting and efficacy of LNPs.

## Figures and Tables

**Table 1:** Currently ongoing clinical trials with LNP systems as per clinicaltrials.gov.

NCT Number	Conditions	Interventions	Phase	Sponsor/Collaborators	Drug Type
NCT03823040	Tinea	Oxiconazole Nitrate Cream 1% Oxiconazole nitrate SLN-loaded gel.	I	Minia University	Small Molecule in Solid LNP
NCT05639894	Respiratory Syncytial Virus Infection	Dose Ranging with RSV mRNA LNP CL-0059 RSV mRNA LNP CL-0137 LNP CL-0137 High Dose Other: Placebo	I/II	Sanofi	mRNA Vaccine in LNPs CL-0059 and CL-137
NCT05267899	Advanced Solid Tumors	WGI-0301	I	Zhejiang Haichang Biotech Co., Ltd.	ASO in LNP
NCT05040373	Hereditary Transthyretin- mediated (hATTR) Amyloidosis Polyneuropathy	Patisiran–LNP in pregnant women	-	Alnylam Pharmaceuticals	Onpattro^®^; RNAi encapsulated in LNP
NCT03739931	Refractory Solid Tumor Malignancies or Lymphoma, Triple Negative Breast Cancer, HNSCC, Non-Hodgkin Lymphoma, Urothelial Cancer, Immune Checkpoint Refractory Melanoma, and NSCLC	mRNA-2752 Durvalumab	I	ModernaTX, Inc. AstraZeneca	Combination Therapeutics with LNP-encapsulated mRNA along with Monoclonal Antibodies
NCT04283461	COVID-19 Immunization	mRNA-1273	I	NIAID ModernaTX, Inc.	mRNA Vaccine in LNP
NCT03323398	Refractory Solid Tumor Malignancies or Lymphoma Ovarian Cancer	mRNA-2416 Durvalumab	I/II	ModernaTX, Inc.	Combination therapy of LNP encapsulated mRNA in combination with mAb
NCT02314052	Hepatocellular Carcinoma	DCR-MYC	I/II	Dicerna Pharmaceuticals, Inc.	siRNA formulated in LNP
NCT04785144	COVID-19 Immunization	mRNA-1273 mRNA-1273.351	I	NIAID ModernaTX, Inc.	mRNA Vaccine in LNP
NCT01858935	Healthy	ND-L02-s0201 Injection	I	Bristol-Myers Squibb Nitto Denko Corporation	Vitamin A-coupled LNP encapsulating siRNA
NCT01437007	Hepatic Metastases involving Pancreas Cancer, Gastric Cancer, Breast Cancer, Ovarian Cancer, and Colorectal Cancer	TKM-080301	I	National Cancer Institute (NCI) National Institutes of Health Clinical Center (CC)	LNP containing siRNA
NCT02110563	Solid Tumors, Multiple Myeloma, Non-Hodgkin Lymphoma, Pancreatic Neuroendocrine Tumors, PNET, NHL	DCR-MYC	I	Dicerna Pharmaceuticals, Inc.	siRNA formulated in LNP
NCT02227459	Hepatic Fibrosis (METAVIR F3-4)	ND-L02-s0201 Injection	I	Bristol-Myers Squibb Nitto Denko Corporation	Vitamin A-coupled LNP containing siRNA
NCT05057182	COVID-19 Vaccine	BNT162b2	IV	The University of Hong Kong	mRNA Vaccine in LNP
NCT04803500	Immediate Implant Placement, Bone Regeneration, Alveolar Bone Resorption	Simvastatin	II	Ain Shams University	Small Molecule in Solid LNP
NCT05062980	Non-Small Cell Lung Cancer	quaratusugene ozeplasmid pembrolizumab docetaxel ramucirumab	I/II	Genprex, Inc.	Combination Therapy of DNA plasmid encapsulated in LNP with mAb
NCT04486833	Carcinoma, Non-Small Cell Lung Cancer	quaratusugene ozeplasmid osimertinib	I/II	Genprex, Inc.	Combination Therapy of DNA plasmid encapsulated in LNP with mAb
NCT05581641	Malaria	BNT165b1	I	BioNTech SE	RNA encapsulated in LNP
NCT04758962	Virus Diseases	1 µg CoV2 SAM (LNP)	I	GlaxoSmithKline	Self-amplifying mRNA vaccine in LNP
NCT05658523	COVID-19	Bivalent Moderna Novavax	III	MCRI, CEPI and PDIII	mRNA Vaccine encapsulated in LNP
NCT01960348	TTR-mediated Amyloidosis	patisiran (ALN-TTR02) Sterile Normal Saline (0.9% NaCl)	III	Alnylam Pharmaceuticals	Onpattro^®^; RNAi encapsulated in LNP
NCT04616872	Atherosclerosis Coronary Artery Disease	Methotrexate-LDE	II/III	University of Sao Paulo General Hospital	Methotrexate in a lipid nanoparticle (MTX-LDE)
NCT04816669	COVID-19	BNT162b2	III	BioNTech SE Pfizer	mRNA Vaccine in LNP
NCT04566276	COVID-19 Vaccine Safety Issues	ChulaCov19 vaccine	I/II	ChulaVRC; CEVRD; Chula CRC; KCMH; CEVT; NVIT in Thailand	mRNA Vaccine encapsulated in LNP
NCT04035525	Healthy	Dietary Supplement: MaxSimil^®^ fish oil + CoQ10 Dietary Supplement: Rice bran oil + CoQ10 Dietary Supplement: CoQ10 as powder form	Not Applicable	Mélanie Plourde Université de Sherbrooke	Small Molecule encapsulated in various carriers including but not limited to SLN and nanostructured lipid carriers
NCT05231369	COVID-19	ChulaCov19 BNA159 mRNA vaccine	I	Chula CRC, ACRO	mRNA Vaccine encapsulated in LNP
NCT04601051	Hereditary Transthyretin Amyloidosis with Polyneuropathy (ATTRv-PN) and Transthyretin Amyloidosis-Related Cardiomyopathy (ATTR-CM)	NTLA-2001	I	Intellia Therapeutics	(CRISPR)/Cas9 gene editing system delivered by lipid nanoparticles
NCT04610567	Coronavirus Inflammation COVID-19	Methotrexate-LDE phase 1 Methotrexate-LDE phase 2	I/II	USPGM; HSM	Methotrexate in LNP (MTX-LDE)
NCT04811664	SARS-CoV-2 Infection	Moderna COVID-19 Vaccine	III	NIAID	mRNA Vaccine in LNP
NCT04675996	Solid Tumor	INT-1B3	I	InteRNA	LNP formulated microRNA
NCT05516459	Coronavirus Infections	Pfizer BNT162b2 Vaccine		SUMC Negev	mRNA Vaccine in LNP
NCT05526066	Ornithine Transcarbamylase Deficiency	ARCT-810	II	Arcturus Therapeutics, Inc.	mRNA formulated in LNP
NCT04416126	Ornithine Transcarbamylase Deficiency	ARCT-810	I	Arcturus Therapeutics, Inc.	mRNA formulated in LNP
NCT05712538	Cystic Fibrosis	ARCT-032 Placebo	I	Arcturus Therapeutics, Inc., Novotech CRO	mRNA formulated in LNP
NCT04442347	Ornithine Transcarbamylase Deficiency	ARCT-810	I	Arcturus Therapeutics, Inc.	mRNA formulated in LNP
NCT05534048	SARS-CoV-2 Infection	PTX-COVID19-B Comirnaty^®^	III	Everest Medicines (Singapore) Pte. Ltd.	mRNA Vaccine in LNP
NCT05534035	SARS-CoV-2 Infection	PTX-COVID19-B Vaxzevria^®^	III	Everest Medicines (Singapore) Pte. Ltd.	mRNA Vaccine in LNP
NCT05525208	COVID-19	SARS-CoV-2 subunit protein recombinant vaccine Active Comparator	II	PT Bio Farma Universitas Padjadjaran Udayana University	mRNA Vaccine in LNP
NCT04847102	COVID-19 SARS-CoV-2	SARS-CoV-2 mRNA Vaccine	III	Walvax Biotechnology, Abogen Biosciences, Yuxi Walvax Biotechnology	mRNA Vaccine in LNP
NCT05057169	COVID-19 Vaccination	BNT162b2 CoronaVac	IV	The University of Hong Kong	mRNA Vaccine in LNP
NCT05168813	SARS-CoV-2 HIV	Moderna mRNA-1273 Vaccine 3 Dose Vaccine 2 Dose	II/III	COVID-19 Prevention Network, NIAID MRC, South Africa	mRNA Vaccine encapsulated in LNP
NCT05755620	Influenza	Influenza Virus Quadrivalent Inactivated Vaccine VRC- FLUNPF099-00-VP (H1ssF_3928)	I	NIAID	mRNA Vaccine in LNP
NCT05497453	Hepatocellular Carcinoma and other solid tumors known for association with MYC Oncogene (MYCHELANGELO I)	OTX-2002 Tyrosine kinase inhibitor One Tyrosine kinase inhibitor Two Checkpoint Inhibitor, Immune	I/II	Omega Therapeutics	Bicistronic mRNA encapsulated in LNP
NCT04776317	COVID-19	ChAdV68-S; ChAdV68-S-TCE; SAM-LNP-S; SAM-LNP-S-TCE	I	NIAID Gritstone bio, Inc.	mRNA Vaccine in LNP
NCT04889209	COVID-19	Ad26.COV2.S; cBNT162b2; mRNA-1273; mRNA-1273.211; mRNA-1273.222; SARS-CoV-2 rS/M1	I/II	NIAID	mRNA Vaccine in LNP
NCT04480957	SARS-CoV-2	ARCT-021	I/II	Arcturus Therapeutics, Inc.	mRNA Vaccine in LNP
NCT05289037	COVID-19	AS03; BNT162b2; BNT162b2 (B.1.1.529) BNT162b2 (B.1.351) BNT162b2 bivalent (wildtype and Omicron BA.1) BNT162b2 bivalent (wildtype and Omicron BA.4/BA.5) CoV2 preS dTM [B.1.351] CoV2 preS dTM/D614 CoV2 preS dTM/D614+B.1.351 mRNA-1273 and 4 more	I/II	NIAID	mRNA Vaccine in LNP
NCT05387317	COVID-19	Pfizer-BioNTech Standard dose AstraZeneca Standard dose Pfizer-BioNTech Fractional dose AstraZeneca Fractional dose Moderna Standard dose Moderna Fractional dose	III	MCRI; CEPI; PDIII; Universitas Padjadjaran Health Development Policy Agency, Ministry of Health Republic of Indonesia	mRNA Vaccine in LNP
NCT05703971	Small Cell Lung Cancer Extensive Stage	quaratusugene ozeplasmid atezolizumab	I/II	Genprex Inc.	Combination Therapy of DNA plasmid encapsulated in LNP with mAb
NCT05543356	COVID-19	Tozinameran; Elasomeran; Bivalent Pfizer- BioNTech; Bivalent Moderna	III	MCRI; CEPI; PDIII	mRNA Vaccine in LNP
NCT05095727	Glycogen Storage Disease	mRNA-3745	I	ModernaTX Inc.	mRNA in LNP
NCT04821674	COVID-19	DS-5670a	I/II	Daiichi Sankyo	mRNA Vaccine in LNP
NCT04844268	SARS-CoV-2	VACCINE RNA MCTI CIMATEC HDT (HDT-301)	I	SENAI CIMATEC	mRNA Vaccine in LNP

Abbreviations: National Institute of Allergy and Infectious Diseases = NIAID; Monoclonal Antibody = mAb; Murdoch Childrens Research Institute = MCRI, Coalition for Epidemic Preparedness Innovations = CEPI; The Peter Doherty Institute for Infection and Immunity = PDIII; Chulalongkorn University Chula Vaccine Research Center, Bangkok, Thailand = ChulaVRC; Center of Excellence in Vaccine Research and Development, Faculty of Medicine, Chulalongkorn University, Bangkok, Thailand = CEVRD; Chula Clinical Research Center, King Chulalongkorn Memorial Hospital, Faculty of Medicine, Chulalongkorn University, Bangkok, Thailand = Chula CRC; King Chulalongkorn Memorial Hospital, Faculty of Medicine, Chulalongkorn University, Bangkok, Thailand = KCMH; Center of Excellence for Vaccine Trial (Vaccine Trial Centre), Faculty of Tropical Medicine, Mahidol University, Bangkok, Thailand = CEVT; National Vaccine Institute, Thailand = NVIT; Academic Clinical Research Office, Faculty of Medicine, Khon Kaen University BioNet-Asia= ACRO; University of Sao Paulo General Hospital = USPGM; Hospital Santa Marcelina = HSM; Soroka University Medical Center Ben-Gurion University of the Negev = SUMCs.

**Table 2:** Constituent lipid information on approved RNA LNP products.

Product Name	Sponsor	Ionizable Lipid	Structural Lipid	pKa of Ionizable Lipid	Ref.
Onpattro	Alnylam Pharmaceuticals, Inc.	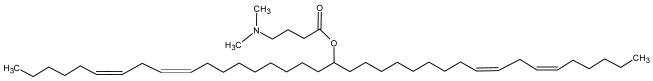 DLin-MC3-DMA	Cholesterol, DSPC, PEG2000-C-DMG	6.35	[5]
Comirnaty	Pfizer Inc.	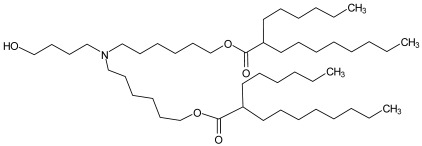 ALC-0315	Cholesterol, DSPC, ALC-0159	6.09	[5]
Spikevax	Moderna	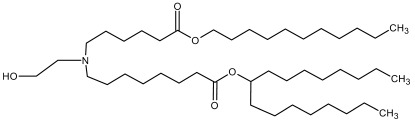 SM-102/Lipid-H	Cholesterol, DSPC, PEG2000-DMG	6.68	[6]

Abbreviations: DLin-MC3-DMA: (6Z,9Z,28Z,31Z)-heptatriacont-6,9,28,31-tetraene-19-yl 4-(dimethylamino)butanoate; DSPC: 1,2-distearoyl-sn-glycero-3-phosphocholine; PEG2000-C-DMG: 1,2-dimyristoyl-rac-glycero-3-carbonylaminoethyl-ω-methoxypolyethylene glycol-2000; ALC-0315: (4-hydroxybutyl) azanediyl) bis (hexane-6,1-diyl) bis (2-hexyldecanoate); ALC-0159: 2-(polyethylene glycol 2000)-N,N-ditetradecylacetamide; PEG2000-DMG: 1,2-dimyristoyl-rac-glycero-3-methoxypolyethylene glycol-2000.

**Table 3:** Commonly used pH-sensitive linkers and their structures.

pH-Sensitive Linker	Chemical Structure
Trans-2-aminocylohexenol (TACH)	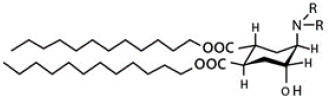
Acetal Group	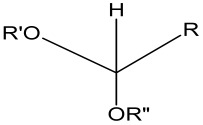
Vinyl Ether Group	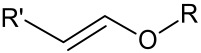
Ortho ester Group	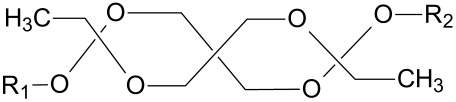
Hydrazone Group	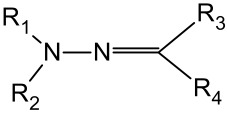

## Data Availability

Not applicable.
